# Fibroblast activation protein activated antifibrotic peptide delivery attenuates fibrosis in mouse models of liver fibrosis

**DOI:** 10.1038/s41467-022-29186-8

**Published:** 2022-03-21

**Authors:** Jaiwoo Lee, Junho Byun, Gayong Shim, Yu-Kyoung Oh

**Affiliations:** 1 College of Pharmacy and Research Institute of Pharmaceutical Sciences, Seoul National University, 1 Gwanak-ro, Gwanak-gu, Seoul, 08826 Republic of Korea; 2 School of Systems Biomedical Science and Integrative Institute of Basic Sciences, Soongsil University, Seoul, 06978 Republic of Korea

**Keywords:** Biomaterials - proteins, Drug delivery, Drug delivery, Liver fibrosis

## Abstract

In liver fibrosis, activated hepatic stellate cells are known to overexpress fibroblast activation protein. Here we report a targeted antifibrotic peptide-delivery system in which fibroblast activation protein, which is overexpressed in fibrotic regions of the liver, liberates the antifibrotic peptide melittin by cleaving a fibroblast activation protein-specific site in the peptide. The promelittin peptide is linked to pegylated and maleimide-functionalized liposomes, resulting in promelittin-modified liposomes. The promelittin-modified liposomes were effective in reducing the viability of activated hepatic stellate cells but not that of control cells. In three types of liver fibrosis mouse models, intravenously administered promelittin-modified liposomes significantly reduces fibrotic regions. In addition, in the bile duct ligation mouse model promelittin-modified liposome-treatment increases overall survival. Although this peptide-delivery concept was tested for liver fibrosis, it can potentially be adapted to other fibrotic diseases.

## Abstract (web summary)

Activated hepatic stellate cells contribute towards the pathogenesis of liver fibrosis, and overexpress fibroblast activation protein. Here the authors report a targeted peptide-delivery system in which fibroblast activation protein liberates the antifibrotic peptide melittin, and demonstrate the approach attenuates fibrosis in mouse models of liver fibrosis.

## Introduction

Liver fibrosis is an incurable condition that afflicts millions of patients globally^1^. Cirrhosis, the most common cause of liver fibrosis, is responsible for over 1 million deaths per year^2^. Despite the clear clinical need, therapies for liver fibrosis are limited. Some drugs, including selonsertib, cenicriviroc and simtuzumab, are in clinical trials, but none has yet been approved for the treatment of liver fibrosis^3^. Among the various cell types in the fibrotic liver, hepatic stellate cells play a central role in the progression of hepatic fibrogenesis. In response to liver injury, quiescent hepatic stellate cells transdifferentiate into activated hepatic stellate cells (aHSC), which possess fibrogenic properties^4^. aHSC are emerging as therapeutic targets for novel therapies against liver fibrosis^5^. Indeed, liver fibrosis can be ameliorated by reducing the aHSC population or inducing aHSC senescence. Nilotinib, which has been investigated for liver fibrosis therapy, is known to induce autophagic cell death and apoptosis of aHSC^6^. OSU-03012, a celecoxib derivative, has been investigated for its potential to induce senescence of aHSC and promote regression of liver fibrosis^7^.

Fibroblast activation protein (FAP), a type II transmembrane glycoprotein, is known to be specifically overexpressed on the surfaces of aHSC in the fibrotic liver. In fact, it has been reported that FAP is exclusively overexpressed in aHSC^8–10^. The active site of FAP, located in an extracellular portion of the protein, cleaves a Pro-Xxx sequence in amino acid bonds. If FAP were used to activate antifibrotic therapy and shown to subsequently reduce the population of abnormally high aHSC in the fibrotic liver, the therapy should be fibrosis-specific.

The naturally occurring peptide melittin has been reported to have therapeutic potential by virtue of its antifibrotic properties^11,12^. Melittin, the principal peptide component of the venom of the honeybee, *Apis mellifera*, exhibits strong, non-specific lytic activity against lipid components of cell membranes. Although melittin can be effective in reducing aHSC populations in fibrotic liver tissues, clinical application of melittin against fibrotic diseases has been limited owing to its non-specific actions, which result in systemic toxicity. A specific activation system that enabled melittin to act only in fibrotic tissue would represent a significant repurposing of melittin.

In this study, we tested the hypothesis that FAP-selective liberation of the antifibrotic peptide, melittin, on the surfaces of FAP-overexpressing aHSC provides a targeted antifibrotic effect in the liver. To this end, we designed a delivery system in which promelittin is cleaved only in FAP-expressing (i.e., fibrotic) regions of the liver, resulting in melittin release into the fibrotic liver microenvironment. To enhance liver delivery and stability in the bloodstream, we further tethered promelittin onto the surfaces of PEGylated liposomes. Here, we report the antifibrotic and survival-prolonging effects of promelittin-tethered liposomes in three different in vivo liver fibrosis mouse models: the bile duct-ligation (BDL), carbon tetrachloride-induced, and high-fat diet-induced models.

## Results

### FAP-specific activation of promellitin-modified liposomes (PRL)

**Fig. 1:**
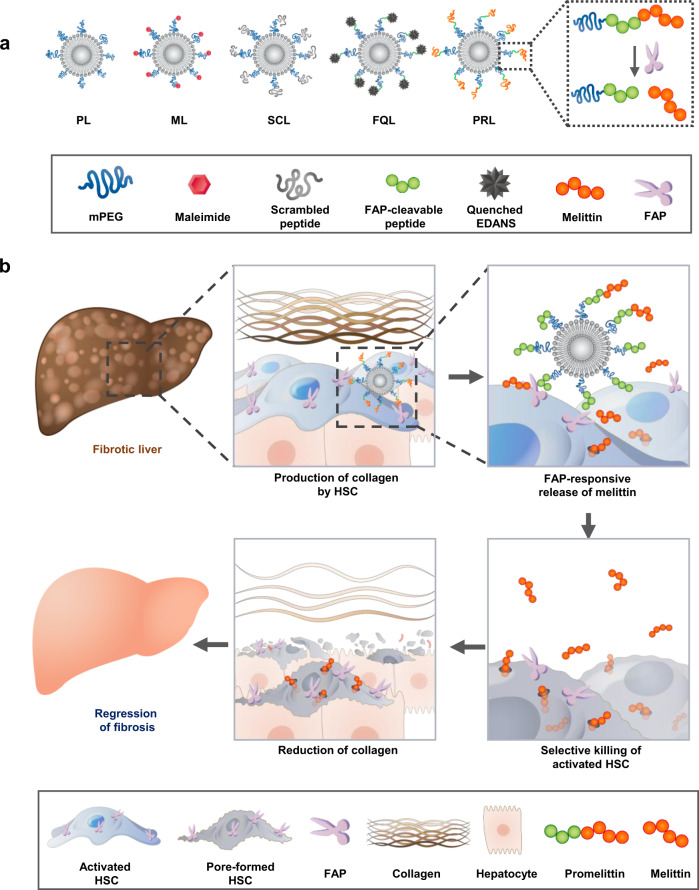
Proposed working mechanism of PRL against liver fibrosis. **a** Schematic illustration of various liposome formulations. FAP-responsive PRL were synthesized by linking cys-promelittin onto the surface of ML. **b** In a fibrotic microenvironment, the promelittin moiety on PRL is cleaved by FAP on aHSC. FAP-mediated cleavage of promelittin on PRL releases melittin, which diffuses to nearby aHSC, creating pores in their cell membranes. The resulting reduction in the number of aHSC in fibrotic tissues leads to decreased production of collagens and regression of fibrosis. PL plain PEGylated liposomes; ML maleimide-activated PEGylated liposomes; SCL scrambled cys-promelittin peptide-tagged liposomes; FQL promelittin-mimicking fluorescence-quenched peptide-tagged liposomes; PRL promelittin-modified liposomes; FAP fibroblast activation protein; EDANS, 5-[(2-aminoethyl) amino]naphthalene-1-sulfonic acid; HSC hepatic stellate cell.

PRL and various other liposome formulations, including plain PEGylated liposomes (PL), maleimide-activated PEGylated liposome (ML), scrambled cys-promelittin peptide-tagged liposomes (SCL) and promelittin-mimicking fluorescence-quenched peptide-tagged liposomes (FQL) are schematically depicted in Fig. 1a. PRL were constructed by tethering cys-promelittin in which a cysteine residue was added to the N-terminus of promelittin to the surface of ML (Fig. 1a). Promelittin has a FAP-cleavable peptide sequence located at the N-terminus of mature melittin. The proposed working mechanism of PRL is illustrated in Fig. 1b. Promelittin in PRL is cleaved by FAP on aHSC, releasing melittin. The liberated melittin creates pores in nearby aHSC, thereby reducing the aHSC population in fibrotic tissues and ultimately resulting in a decrease in collagen fibers and regression of fibrosis.

**Fig. 2:**
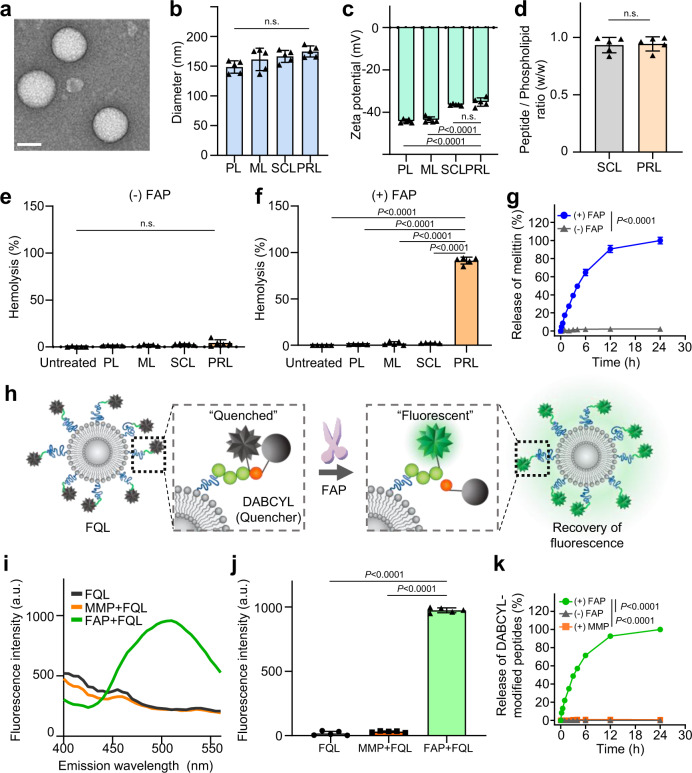
FAP-specific cleavage of promelittin peptide on PRL. **a** Transmission electron microscopy (TEM) imaging. TEM images of liposomes, obtained using a Talos L120C TEM system operating at 120 kV. Scale bar: 100 nm. **b** Size measurements. Liposomes size was measured using dynamic light scattering (*n* = 5 independent samples per group). Significant differences were assessed using a one-way ANOVA with Tukey test (n.s.: *P* > 0.05). Results are presented as mean ± S.D. **c** Zeta potential measurements. Zeta potential of liposomes was measured by electrophoretic light scattering (*n* = 5 independent samples per group). Significant differences were assessed using a one-way ANOVA with Tukey test (Tukey’s test *P*_PRL–__PL_ < 0.0001, *P*_PRL–__ML_ < 0.0001, *P*_PRL-SCL_ > 0.05). Results are presented as mean ± S.D. **d** Peptide-to-phospholipid ratios were estimated using fluorescamine and phosphate assays (*n* = 5 independent samples per group). Significant differences were assessed using a one-way ANOVA with Tukey test (n.s.: *P* > 0.05). Results are presented as mean ± S.D. **e**, **f** The hemolytic activity of PRL against mouse red blood cells (RBCs) was evaluated using hemolysis assays (*n* = 5 independent samples per group). Significant differences were assessed using a one-way ANOVA with Tukey test (Tukey’s test (−) FAP; n.s.: *P* > 0.05, (+) FAP: *P*_PRL-untreated_ < 0.0001, *P*_PRL-PL_ < 0.0001, *P*_PRL-ML_ < 0.0001, *P*_PRL-SCL_ < 0.0001). Results are presented as mean ± S.D. **g** Release kinetics of melittin from PRL. PRL was incubated with or without FAP for various periods. The release of melittin at each point was measured by fluorescamine assay (*n* = 5 independent samples per group). Significant differences were assessed using a one-way ANOVA with Tukey test (Tukey’s test *P*_(+)FAP - (−)FAP_ < 0.0001). Results are presented as mean ± S.D. **h** Design of promelittin-mimicking fluorescence-quenched peptide-tagged liposomes (FQL). A fluorescent FRET (fluorescence resonance energy transfer) moiety was attached to the promelittin-mimicking peptide containing a FAP-dependent cleavage site, and the FAP-specific activity of the peptide was determined by assessing FAP-responsive fluorescence. **i** Fluorescence emission spectra of FQL observed after FAP or MMP treatment. **j** Mean fluorescent intensity of FQL at an emission wavelength of 490 nm (*n* = 5 independent samples per group). Significant differences were assessed using a one-way ANOVA with Tukey test (Tukey’s test *P*_FAP+FQL - FQL_ < 0.0001, *P*_FAP+FQL - MMP+FQL_ < 0.0001). Results are presented as mean ± S.D. **k** Release kinetics of DABCYL-modified peptides from FQL. FQL was incubated in the absence or presence of FAP, or in the presence of MMP. The release of DABCYL-modified peptides was evaluated via spectrofluorometry (*n* = 5 independent samples per group). Significant differences were assessed using a one-way ANOVA with Tukey test (Tukey’s test *P*_(+)FAP - (+)MMP_ <0.0001, *P*_(+)FAP - (−)FAP_ < 0.0001). Results are presented as mean ± S.D. PL plain PEGylated liposomes; ML maleimide-activated PEGylated liposomes; SCL scrambled cys-promelittin peptide-tagged liposomes; FQL promelittin-mimicking fluorescence-quenched peptide-tagged liposomes; PRL promelittin-modified liposomes; h hour; DABCYL 4-((4-(dimethylamino) phenyl)azo)benzoic acid; MMP matrix metalloproteinase. Source data are provided as a Source Data file.

Transmission electron microscopy (TEM) images revealed that PRL adopted a spherical shape (Fig. 2a). Surface modification of ML with cys-promelittin or scrambled peptide did not significantly affect the size of liposomes (Fig. 2b). Zeta potentials of SCL and PRL increased slightly after peptide conjugation (Fig. 2c), but peptide-to-phospholipid ratios did not differ significantly between PRL and SCL (Fig. 2d). The stability of liposomes was not affected by FAP. During the 18-day study period, none of the liposomes showed a significant size change, regardless of FAP or storage temperature (Supplementary Fig. 2). Although the physicochemical features of PRL did not differ from those of SCL, the two formulations differed in their biological responsiveness to the FAP enzyme. In the absence of FAP, there was no hemolysis of RBCs regardless of liposome type (Fig. 2e). However, PRL, but not other liposome formulations, showed RBC hemolysis in the presence of FAP (Fig. 2f). The release of melittin from PRL was observed in the presence of FAP, but not in the absence of FAP (Fig. 2g). In the presence of FAP, the release of melittin from PRL linearly increased up to 12 h of incubation, when it reached 90.7% release. After 24 h of incubation, 99.6% release was observed.

FAP-specific cleavage of promelittin on PRL was further tested by monitoring the recovery of quenched fluorescence of FQL in the presence of FAP. The fluorescence intensity of FQL, dually modified with the fluorophore EDANS and quencher DABCYL, is quenched by FRET. Cleavage of promelittin by FAP disrupts the EDANS and DABCYL interaction and recovers the fluorescence of EDANS (Fig. 2h). Treatment with matrix metalloproteinase (MMP) did not significant change the fluorescence intensity of FQL (Fig. 2i). In contrast, FAP treatment substantially enhanced FQL fluorescence, increasing the fluorescence intensity of FQL at 490 nm by 31.3-fold compared with no-FAP controls (Fig. 2j). The release of fluorescent melittin from FQL was observed in the presence of FAP, but not in the absence of FAP or in the presence of MMP (Fig. 2k). In the presence of FAP, the release of fluorescent melittin gradually increased with time. More than 50% release was observed within 4 h of incubation with FAP. After 12 h of incubation, 92.6% of the fluorescent melittin had been liberated from FQL.

### FAP-specific responsiveness of PRL to aHSC

**Fig. 3:**
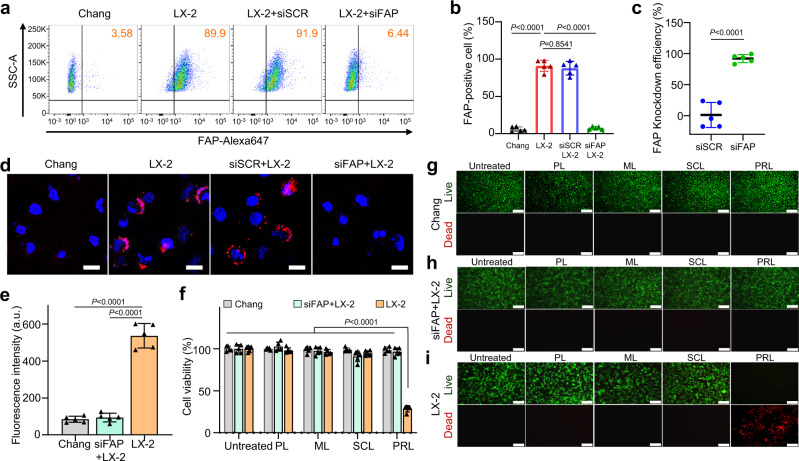
Viability of normal and hepatic stellate cells after treatment with various liposome formulations. **a** LX-2 cells, Chang cells, and siSCR or siFAP-treated LX-2 cells were stained with rabbit anti-FAP primary antibody and FITC-conjugated anti-rabbit secondary antibody. Fluorescence-positive cells were determined by flow cytometry (*n* = 5 biologically independent samples per group). **b** FAP positive cell population was analyzed between LX-2 cells, Chang cells, and siSCR or siFAP-treated LX-2. Significant differences were assessed using a one-way ANOVA with Tukey test (Tukey’s test *P*_LX-2 - Chang_ < 0.0001, *P*_LX-2 - siFAP LX-2_ < 0.0001, P_LX-2 - siSCR LX-2_ < 0.0001). Results are presented as mean ± S.D. **c** The efficiency of the siFAP in LX-2 cells was evaluated compare to siSCR. Significant differences were assessed using a one-way ANOVA with Tukey test (Tukey’s test *P*_siFAP - siSCR_ < 0.0001). Results are presented as mean ± S.D. **d** Representative confocal images evaluating the efficiency of siFAP in LX-2 cells. Red color indicates FAP expression. **e** The fluorescence emission intensity of FQL was measured at a wavelength of 490 nm after incubation with LX-2 cells, Chang cells, or siFAP-treated LX-2 cells (*n* = 5 biologically independent samples per group). Significant differences were assessed using a one-way ANOVA with Tukey test (Tukey’s test *P*_LX-2 - Chang_ < 0.0001, *P*_LX-2 - siFAP LX-2_ < 0.0001). Results are presented as mean ± S.D. **f** MTT assays were used to determine cell viability of following treatment with various liposome formulations (*n* = 5 biologically independent samples per group). Significant differences were assessed using a one-way ANOVA with Tukey test (Tukey’s test *P*_PRL treated LX-2 - others_ < 0.0001). Results are presented as mean ± S.D. **g**–**i** Fluorescence microscopy assessment of live (green)/dead (red) Chang cells (**g**), siFAP-treated LX-2 cells (**h**), and LX-2 cells (**i**). Scale bar: 50 μm. PL plain PEGylated liposomes; ML maleimide-activated PEGylated liposomes; SCL scrambled cys-promelittin peptide-tagged liposomes; PRL promelittin-modified liposomes. Source data are provided as a Source Data file.

Having demonstrated the responsiveness of PRL to FAP in a cell-free setting, we next assessed PRL responsiveness to aHSC using the human LX-2 hepatic stellate cell line as a model system. In these experiments, LX-2 cells or Chang cells, used as a FAP-negative control cell, were treated with various liposome formulations, and cleavage of liposomal promelittin by FAP on cells was assessed by monitoring recovery of quenched EDANS fluorescence. Flow cytometry data showed that the treatment of LX-2 cells with FAP-specific siRNA (siFAP) significantly decreased the expression of FAP (Fig. 3a, b). In siFAP-treated cells, the expression level of FAP was silenced by 96.3% compared to that in untreated cells (Fig. 3c). Confocal imaging was used to further visualize the silencing of FAP protein in LX-2 cells (Fig. 3d). Following treatment with FQL, Chang cells showed no change in fluorescence intensity, whereas siFAP-pretreated LX-2 cells showed little change in fluorescence. In contrast, treatment of LX-2 cells with FQL induced a substantial increase in fluorescence (Fig. 3e), indicating effective cleavage of promelittin and recovery of EDANS fluorescence.

Using MTT assays to assess cell viability following treatment with various liposome formulations, we found that PL, ML, and SCL had no effect on the viability of Chang cells, LX-2 cells, or siFAP-treated LX-2 cells (Fig. 3f). Although PRL also had no significant effect on the viability of Chang cells or siFAP-treated LX-2 cells, they significantly affected the viability of LX-2 cells, reducing it to less than 40% (Fig. 3f). Fluorescence live/dead cell staining confirmed these effects, showing that Chang cells (Fig. 3g) and siFAP-treated LX-2 cells (Fig. 3h) retained full viability follow treatment with liposomes, regardless of liposome type, whereas only treatment of LX-2 cells with PRL resulted in an increase in the dead cell population (Fig. 3i).

### FAP expression and activation of PRL in a liver fibrosis model

**Fig. 4:**
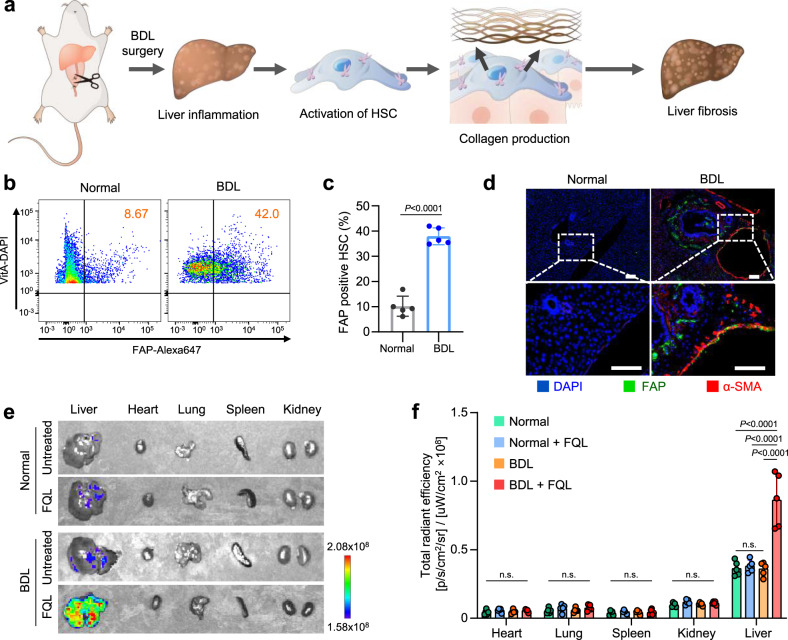
FAP-mediated cleavage of liposomal promelittin in BDL induced fibrosis mice. **a** Experimental scheme for induction of liver fibrosis using the BDL method (*n* = 5 biologically independent mice per group). **b**, **c** FAP expression level in HSC from normal mice or BDL mice was analyzed by flow cytometry. (*n* = 5 biologically independent samples per group). Significant differences were assessed using a one-way ANOVA with Tukey test (Tukey’s test *P*_BDL - Normal_ < 0.0001). Results are presented as mean ± S.D. **d** Immunohistochemical staining for FAP in liver tissues from normal and BDL mice (*n* = 5 biologically independent samples per group). Scale bar: 50 μm. **e** One hour after mice were intravenously injected with FQL, the fluorescence intensities of vital organs (liver, heart, lung, spleen, kidney) from normal and BDL mice were monitored using an IVIS Spectrum instrument equipped with excitation wavelength at 335 nm and emission wavelength at 490 nm. Living image (v4.5.2., PerkinElmer) was used to analyze fluorescent images of mice. The color bar indicates fluorescence intensity (*n* = 5 biologically independent samples per group). **f** Fluorescence intensity of each organ was calculated between normal and BDL mice. Significant differences were assessed using a one-way ANOVA with Tukey test (Tukey’s test n.s.: *P* > 0.05, Liver: *P*_BDL + FQL - Normal_ < 0.0001, *P*_BDL+FQL - Normal+FQL_ < 0.0001, *P*_BDL+FQL - BDL_ < 0.0001). Results are presented as mean ± S.D. BDL bile duct ligation; αSMA alpha-smooth muscle actin; HSC hepatic stellate cell; PL plain PEGylated liposomes; ML maleimide-activated PEGylated liposomes; SCL scrambled cys-promelittin peptide-tagged liposomes; FQL promelittin-mimicking fluorescence-quenched peptide-tagged liposomes; PRL promelittin-modified liposomes. Source data are provided as a Source Data file.

We next tested whether expression of FAP was increased in a liver fibrosis model, and if so, whether promelittin on PRL could be cleaved in the liver. A BDL liver fibrosis model was established using the scheme illustrated in Fig. 4a. Flow cytometry showed higher expression of FAP in aHSC from the BDL liver compared with cells from a normal liver (Fig. 4b, c). Immunohistochemical staining was performed to assess FAP expression in liver tissue from normal and BDL mice (Fig. 4d). Molecular imaging revealed that PRL was specifically activated at the liver in the BDL model. In normal mice, there was no significant difference of fluorescence intensity regardless of FQL treatment in all organs tested (Fig. 4e, f). In BDL model mice, no significant difference was observed in the heart, lung, spleen, or kidney (Fig. 4e, f). In the liver tissue of BDL model mice, in contrast, a significant difference was observed in the FQL-treated group, which showed 2.5-fold higher fluorescence intensity.

### In vivo antifibrotic effect

**Fig. 5:**
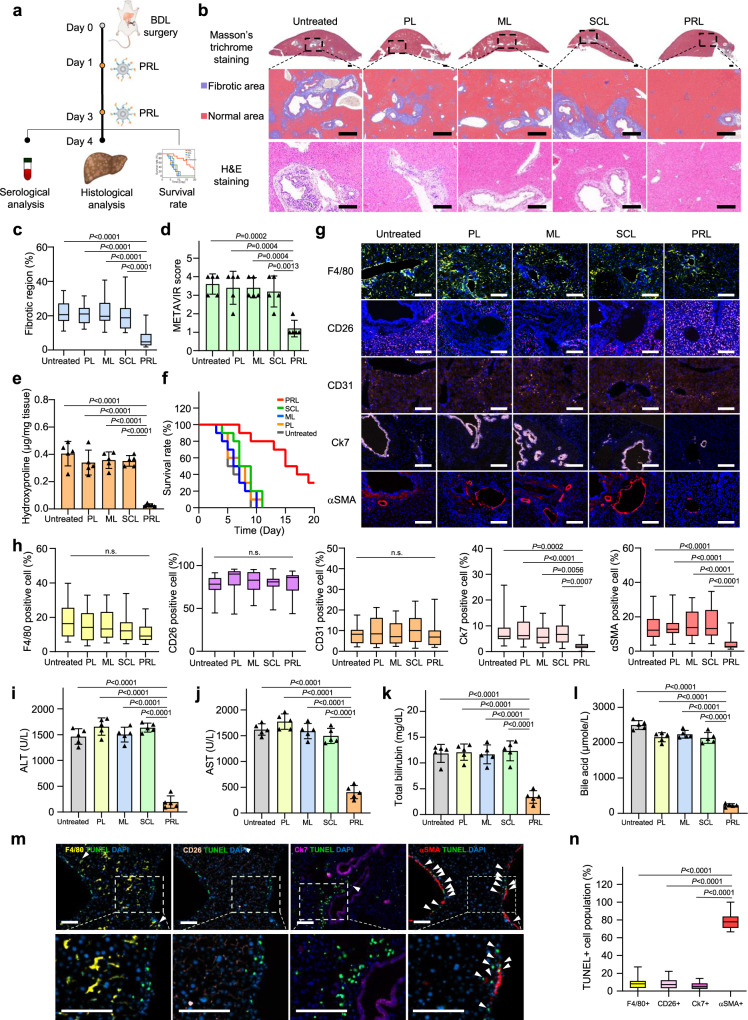
In vivo antifibrotic effects of various liposome preparations in the BDL induced lifer fibrosis model. **a** On days 1 and 3 after BDL surgery, mice were intravenously administered 1 mg/kg PL, ML, SCL, or PRL. On day 4, liver tissues were extracted and further analyzed. **b** Top panel shows representative Masson’s trichrome-staining in BDL induced fibrotic liver. Lower panel shows pseudocolored images by InForm 2.2.1 analysis, distinguishing fibrotic regions (blue) from normal tissue (red). Bottom panel shows representative H&E staining in BDL-induced fibrotic liver treated with various liposomes. (*n* = 5 biologically independent mice per group). Scale bar: 50 μm. **c** Quantification of connective tissue area, calculated from whole images of stained liver, as calculated from five randomly selected fields per sample (*n* = 5 biologically independent mice per group). Significant differences were assessed using a one-way ANOVA with Tukey test (Tukey’s test *P*_PRL–untreated_ < 0.0001, *P*_PRL–PL_ < 0.0001, P_PRL–ML_ < 0.0001, *P*_PRL–SCL_ < 0.0001). In box plots, boxes show the 25th–75th percentile with the median, and whiskers show the minimum–maximum. **d** The severity of liver fibrosis for each group, assessed using the METAVIR scoring (*n* = 5 biologically independent mice per group). Significant differences were assessed using a one-way ANOVA with Tukey test (Tukey’s test *P*_PRL–untreated_ = 0.0002, *P*_PRL–PL_ = 0.0004, *P*_PRL–ML_ = 0.0004, *P*_PRL–SCL_ = 0.0013). Results are presented as mean ± S.D. **e** Collagen content of fibrotic liver samples, measured using hydroxyproline assays (*n* = 5 biologically independent mice per group). Significant differences were assessed using a one-way ANOVA with Tukey test (Tukey’s test *P*_PRL–untreated_ < 0.0001, *P*_PRL–PL_ < 0.0001, *P*_PRL–ML_ < 0.0001, *P*_PRL–SCL_ < 0.0001). Results are presented as mean ± S.D. **f** Survival rates of BDL mice after treatment with various liposome formulations (*n* = 10 biologically independent mice per group). **g** BDL-induced mice were treated with various liposomes. One day after the last dose, liver tissues were frozen-sectioned and stained with antibodies against F4/80 (macrophage), CD26 (hepatocyte), CD31 (endothelial cell), Ck7 (cholangiocyte), and αSMA (aHSC). **h** Populations of cells were analyzed using a VECTRA tissue analyzer and the InForm 2.2.1 image analysis software (*n* = 5 biologically independent mice per group). Scale bar: 50 µm. Significant differences were assessed using a one-way ANOVA with Tukey test (Tukey’s test n.s.: *P* > 0.05, Ck7: *P*_PRL–untreated_ = 0.0002, *P*_PRL–PL_ < 0.0001, *P*_PRL–ML_ = 0.0056, *P*_PRL–SCL_ < 0.0007, αSMA: *P*_PRL–untreated_ < 0.0001, *P*_PRL–PL_ < 0.0001, *P*_PRL–ML_ < 0.0001, *P*_PRL–SCL_ < 0.0001). In box plots, boxes show the 25th–75th percentile with the median, and whiskers show the minimum–maximum. On day 4 after BDL surgery, blood was collected for serum analysis of ALT (**i**), AST (**j**), total bilirubin (**k**), and bile acid (**l**) (*n* = 5 biologically independent samples per group). Significant differences were assessed using a one-way ANOVA with Tukey test (Tukey’s test ALT: *P*_PRL–untreated_ < 0.0001, *P*_PRL–PL_ < 0.0001, *P*_PRL–ML_ < 0.0001, *P*_PRL–SCL_ < 0.0001, AST: *P*_PRL–untreated_ < 0.0001, *P*_PRL–PL_ < 0.0001, *P*_PRL–ML_ < 0.0001, *P*_PRL–SCL_ < 0.0001, Total bilirubin: p_PRL–untreated_ < 0.0001, *P*_PRL–PL_ < 0.0001, *P*_PRL–ML_ < 0.0001, *P*_PRL–SCL_ < 0.0001, Bile acid: *P*_PRL–untreated_ < 0.0001, *P*_PRL–PL_ < 0.0001, *P*_PRL–ML_ < 0.0001, *P*_PRL–SCL_ < 0.0001). Results are presented as mean ± S.D. **m** Liver tissues were stained with TUNEL, followed by immune fluorescence staining with antibodies against αSMA, F4/80, or CD26, and final counter staining with DAPI. Representative images of each group (*n* = 5 biologically independent mice per group) are shown. White arrows show TUNEL-positive spots. Scale bar: 50 µm. **n** Analysis of TUNEL-positive cells in five randomly selected fields per sample as calculated from five randomly selected fields per sample (*n* = 5 biologically independent mice per group). Significant differences were assessed using a one-way ANOVA with Tukey test (Tukey’s test *P*_αSMA+ - F4/80+_ < 0.0001, *P*_αSMA+ - CD26+_ < 0.0001, *P*_αSMA+ - Ck7+_ < 0.0001). In box plots, boxes show the 25th–75th percentile with the median, and whiskers show the minimum–maximum. BDL bile duct ligation; PL plain PEGylated liposomes; ML maleimide-activated PEGylated liposomes; SCL scrambled cys-promelittin peptide-tagged liposomes; PRL promelittin-modified liposomes; CD26 cluster of differentiation 26; CD31 cluster of differentiation 31; Ck7 cytokeratin7; αSMA alpha-smooth muscle actin; ALT alanine transaminase; AST aspartate transaminase; TUNEL terminal deoxynucleotidyl transferase dUTP nick end labeling assay. Source data are provided as a Source Data file.

The experimental scheme for assessing the antifibrotic efficacy of various liposome formulations in a BDL animal model is illustrated in Fig. 5a. Antifibrotic efficacy was evaluated using histological, biochemical, and serological analyses. Fibrotic regions of the liver were visualized by Masson’s trichrome method, which stains collagen fibers blue and normal tissues red. Masson’s trichrome staining showed fibrotic regions in the livers of mice treated with ML, PL, or SCL. In contrast, images of whole livers from mice treated with PRL showed fewer fibrotic (blue-stained) regions compared with livers from mice treated with any other liposome formulation (Fig. 5b). Similar to the Masson’s trichrome staining results, hematoxyllin and eosin (H&E) staining revealed tissue damage in the vicinity of blood vessels for groups treated with PL, ML, or SCL, but not PRL (Fig. 5b). A quantitative analysis of images revealed that Masson’s trichrome-stained liver tissues from mice treated with PRL showed significantly fewer fibrotic regions than those from other treatment groups (Fig. 5c). Applying the METAVIR F staging system, we found that mice treated with PRL showed the lowest scores compared with mice treated with PL, ML, or SCL (Fig. 5d). Biochemical assessments of the antifibrotic efficacy of PRL showed that hydroxyproline levels in BDL mice treated with PL, ML, or SCL did not significantly differ from those in untreated mice. However, hydroxyproline levels in PRL-treated BDL mice were 7.2-fold lower than those in untreated mice (Fig. 5e).

We assessed the survival of BDL mice following treatment with various liposome formulations. Among BDL mice treated with various liposome preparations, those treated with PRL showed the highest survival rate. Specifically, PRL-treated mice showed 80% survival 12 d after BDL surgery, a time point at which mice in all other treatment groups had died (Fig. 5f). Regarding toxicity, normal mice receiving repeated intravenous doses of PRL showed complete survival (Supplementary Fig. 3a). All five mice survived for 60 days after four repeated intravenous administrations of PL or PRL (Supplementary Fig. 3b). Moreover, there was no significant difference in body weight among the groups (Supplementary Fig. 3c).

Treatment of BDL mice with PRL altered the cell populations in liver tissues, which were analyzed with markers for macrophages (F4/80), hepatocytes (cluster of differentiation 26, CD26), endothelial cells (cluster of differentiation 31, CD31), cholangiocytes (cytokeratin7, Ck7), and aHSC (alpha-smooth muscle actin, αSMA). Immunofluorescence imaging (Fig. 5g), quantitative image analysis (Fig. 5g), and quantitative real time polymerase chain reaction (qRT-PCR) (Supplementary Fig. 4) showed that the populations of macrophage, hepatocyte, and endothelial cell were not significantly different regardless of treatment. However, the populations of aHSC and cholangiocyte were significantly decreased by PRL treatment (Fig. 5g, h and Supplementary Fig. 4).

We performed serological analyses with BDL induced liver fibrosis model. PL, ML, or SCL treatment had no significant effect on alanine transaminase (ALT) (Fig. 5i), aspartate transaminase (AST) (Fig. 5j), bile acid (Fig. 5k), or total bilirubin (Fig. 5l) serum levels, which were comparable to those in untreated normal mice. However, 4 days after BDL, serum levels of ALT, AST, bile acid and total bilirubin were all significantly lower following PRL treatment compared with untreated mice or mice treated with other liposome formulations.

TUNEL assays revealed that PRL had an aHSC-selective killing effect. TUNEL-positive cell analysis followed by immunohistochemistry (IHC) showed that the highest population of apoptotic cells was observed among αSMA-positive cells (Fig. 5m). Flow cytometry showed that about 78.6% of the apoptotically dying cell populations were aHSC marked with αSMA (Fig. 5n). The population positive for both TUNEL and αSMA was 9.6-fold higher than that positive for both TUNEL and F4/80.

**Fig. 6:**
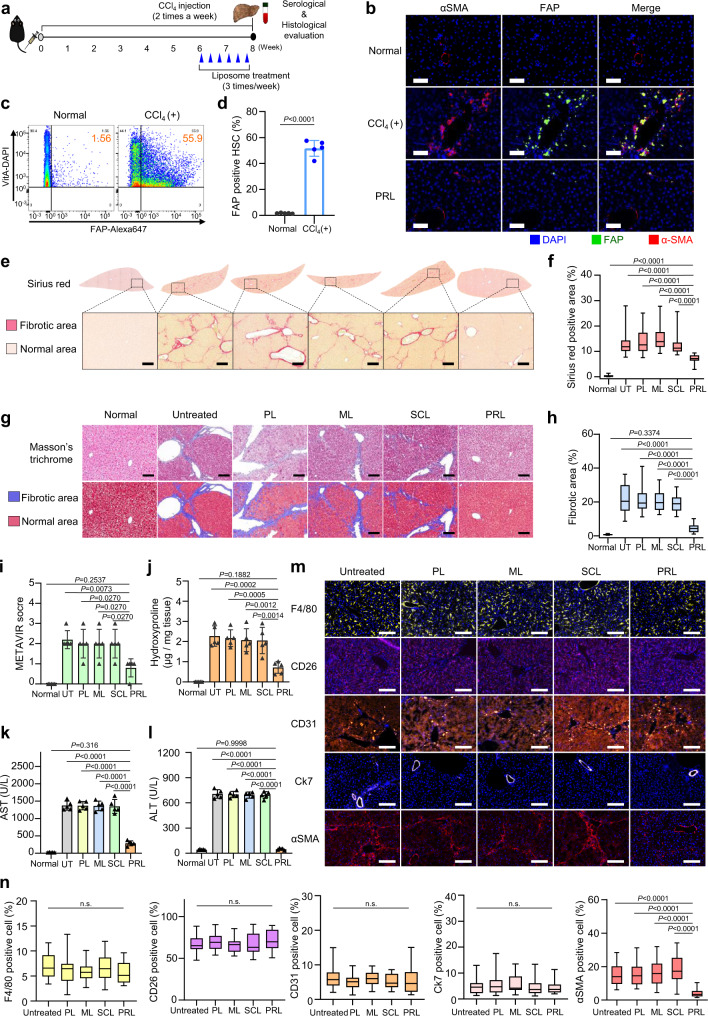
In vivo antifibrotic effects of various liposome preparations in the CCl_4_-induced liver fibrosis model. **a** Schematic of the CCl_4_-induced liver fibrosis model and treatment timelines for the various liposomes. **b** Immunohistochemical staining for FAP in liver tissues from normal and CCl_4_-induced liver fibrosis mice (*n* = 5 biologically independent samples per group). Scale bar: 50 μm. **c**, **d** FAP expression levels on HSCs from normal or CCl_4_-induced fibrotic liver were analyzed by flow cytometry (*n* = 5 biologically independent mice per group). Significant differences were assessed using a one-way ANOVA with Tukey test (Tukey’s test *P*_CCl4(+) - Normal_ < 0.0001). Results are presented as mean ± S.D. **e**, **f** Representative Sirius red-stained liver images for the evaluation of liver fibrosis, and quantification of the Sirius red-positive area (*n* = 5 biologically independent mice per group). Significant differences were assessed using a one-way ANOVA with Tukey test (Tukey’s test *P*_PRL–normal_ < 0.0001, *P*_PRL–untreated_ < 0.0001, *P*_PRL–PL_ < 0.0001, *P*_PRL–ML_ < 0.0001, *P*_PRL–SCL_ < 0.0001). In box plots, boxes show the 25th–75th percentile with the median, and whiskers show the minimum–maximum. **g** Upper panel shows representative Masson’s trichrome-stained images of CCl_4_-induced fibrotic liver. The lower panel displays pseudocolored images by InForm 2.2.1 analysis, distinguishing fibrotic regions (blue) from normal tissue (red) (*n* = 5 biologically independent mice per group). Scale bar: 50 μm. **h** Quantification of the connective tissue area under Masson’s trichrome staining, as calculated from five randomly selected fields per sample (*n* = 5 biologically independent mice per group). Significant differences were assessed using a one-way ANOVA with Tukey test (Tukey’s test *P*_PRL–normal_ = 0.3374, P_PRL–untreated_ < 0.0001, P_PRL–PL_ < 0.0001, *P*_PRL–ML_ < 0.0001, *P*_PRL–SCL_ < 0.0001). In box plots, boxes show the 25th–75th percentile with the median, and whiskers show the minimum–maximum. **i** The severity of liver fibrosis for each group, assessed using the METAVIR scoring system (*n* = 5 biologically independent mice per group). Significant differences were assessed using a one-way ANOVA with Tukey test (Tukey’s test *P*_PRL–normal_ = 0.2537, *P*_PRL–untreated_ = 0.0073, *P*_PRL–PL_ = 0.0270, *P*_PRL–ML_ = 0.0270, *P*_PRL–SCL_ = 0.0270). Results are presented as mean ± S.D. **j** Collagen content of fibrotic liver samples, as measured using hydroxyproline assays. Significant differences were assessed using a one-way ANOVA with Tukey test (Tukey’s test *P*_PRL–normal_ = 0.1882, *P*_PRL–untreated_ = 0.0002, *P*_PRL–PL_ = 0.0005, *P*_PRL–ML_ = 0.0012, *P*_PRL–SCL_ = 0.0014). Results are presented as mean ± S.D. Blood was collected for serum analysis of AST (**k**) and ALT (**l**) (*n* = 5 biologically independent mice per group), UT: untreated group. Significant differences were assessed using a one-way ANOVA with Tukey test (Tukey’s test AST: *P*_PRL–normal_ = 0.3160, *P*_PRL–untreated_ < 0.0001, *P*_PRL–PL_ < 0.0001, *P*_PRL–ML_ < 0.0001, *P*_PRL–SCL_ < 0.0001, ALT: *P*_PRL–normal_ = 0.9998, *P*_PRL–untreated_ < 0.0001, *P*_PRL–PL_ < 0.0001, *P*_PRL–ML_ < 0.0001, *P*_PRL–SCL_ < 0.0001). Results are presented as mean ± S.D. **m** Representative immunofluorescence images of liver tissues stained with various cell markers including F4/80 (macrophage), CD26 (hepatocyte), CD31 (endothelial cell), Ck7 (cholangiocyte), and αSMA (aHSC). **n** Populations of cells were analyzed using a VECTRA tissue analyzer and the InForm 2.2.1 image analysis software (*n* = 5 biologically independent mice per group). Significant differences were assessed using a one-way ANOVA with Tukey test (Tukey’s test n.s.: *P* > 0.05, αSMA: *P*_PRL–untreated_ < 0.0001, *P*_PRL–PL_ < 0.0001, *P*_PRL–ML_ < 0.0001, *P*_PRL–SCL_ < 0.0001). In box plots, boxes show the 25th–75th percentile with the median, and whiskers show the minimum–maximum. Scale bar: 50 µm. HSC hepatic stellate cell; UT untreated group; PL plain PEGylated liposomes; ML maleimide-activated PEGylated liposomes; SCL scrambled cys-promelittin peptide-tagged liposomes; PRL promelittin-modified liposomes; CD26 cluster of differentiation 26; CD31 cluster of differentiation 31; Ck7 cytokeratin7; αSMA alpha-smooth muscle actin; ALT alanine transaminase; AST aspartate transaminase. Source data are provided as a Source Data file.

The antifibrotic efficacy of PRL was evaluated in the CCl_4_-induced chronic liver fibrosis model. The treatment scheme for the CCl_4_-induced liver fibrosis animal model is illustrated in Fig. 6a. Immunohistochemistry (Fig. 6b), FACS analysis (Fig. 6c), and flow cytometry (Fig. 6d) showed that the expression of FAP was higher in mice treated with CCl_4_ over 8 weeks compared to normal mice. Sirius red (Fig. 6e) and Masson’s trichrome (Fig. 6g) staining followed by image analysis (Fig. 6f, h, respectively) showed that the fibrotic regions were significantly decreased in the PRL-treated group compared to the other groups. The in vivo antifibrotic effect of PRL was also evaluated using the METAVIR F staging system (Fig. 6i), which revealed that the METAVIR score was lowest in the PRL-treated group. The level of hydroxyproline, a major component of collagen, was 3.2-fold lower in PRL-treated mice than in untreated mice (Fig. 6j).

Serological analysis revealed that PRL treatment significantly lowered the levels of AST (Fig. 6k) and ALT (Fig. 6l) relative to the other treatments and the untreated fibrotic control. Immunohistochemistry showed that the populations of cells positive for F4/80, CD26, or CD31 were not significantly different among the groups, regardless of treatment, whereas the level of the aHSC marker, αSMA, was significantly lower in the group treated with PRL relative to the other groups (Fig. 6m, n).

**Fig. 7:**
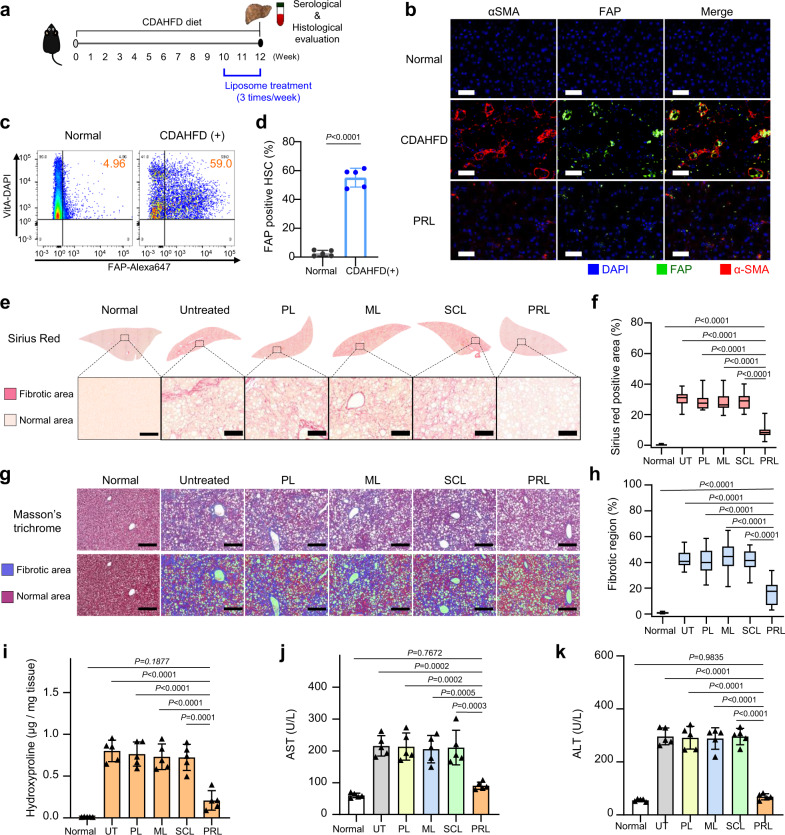
In vivo antifibrotic effects of various liposome preparations in the CDAHFD-induced fibrosis model. **a** Schematic of the CDAHFD-induced fibrosis model and treatment timelines for the various liposomes. **b** Representative images of liver sections stained for FAP (green), αSMA (red), and DAPI (blue) (*n* = 5 biologically independent mice per group). Scale bar: 50 μm. **c**, **d** FAP expression levels on HSCs from normal or CDAHFD-induced fibrotic liver were evaluated by flow cytometry (*n* = 5 biologically independent mice per group). Significant differences were assessed using a one-way ANOVA with Tukey test (Tukey’s test P_CDAHFD(+) - Normal_ < 0.0001). Results are presented as mean ± S.D.. **e**, **f** Representative Sirius red-stained liver images for the evaluation of liver fibrosis and quantification of the Sirius red-positive area (*n* = 5 biologically independent mice per group). Significant differences were assessed using a one-way ANOVA with Tukey test (Tukey’s test *P*_PRL–normal_ < 0.0001, *P*_PRL–untreated_ < 0.0001, *P*_PRL–PL_ < 0.0001, *P*_PRL–ML_ < 0.0001, *P*_PRL–SCL_ <  0.0001). In box plots, boxes show the 25th–75th percentile with the median, and whiskers show the minimum–maximum. **g** Top panel shows representative Masson’s trichrome-staining of liver. Bottom panel displays pseudocolored images by InForm 2.2.1 analysis, distinguishing fibrotic regions (blue) from normal tissue (red) (*n* = 5 biologically independent mice per group). Scale bar: 50 μm. **h** Quantification of Masson’s trichrome-stained connective tissue area calculated from five randomly selected fields per sample (*n* = 5 biologically independent mice per group). Significant differences were assessed using a one-way ANOVA with Tukey test (Tukey’s test *P*_PRL–normal_ < 0.0001, *P*_PRL–untreated_ < 0.0001, *P*_PRL–PL_ < 0.0001, *P*_PRL–ML_ < 0.0001, *P*_PRL–SCL_ < 0.0001). In box plots, boxes show the 25th–75th percentile with the median, and whiskers show the minimum–maximum. **i** Collagen content of fibrotic liver samples, as measured using hydroxyproline assays. Significant differences were assessed using a one-way ANOVA with Tukey test (Tukey’s test *P*_PRL–normal_ = 0.1877, *P*_PRL–untreated_ < 0.0001, *P*_PRL–PL_ < 0.0001, *P*_PRL–ML_ < 0.0001, *P*_PRL–SCL_ = 0.0001). Results are presented as mean ± S.D. Blood was collected for serum analysis of AST (**j**) and ALT (**k**) (*n* = 5 biologically independent mice per group). Significant differences were assessed using a one-way ANOVA with Tukey test (Tukey’s test AST: *P*_PRL–normal_ = 0.7672, *P*_PRL–untreated_ = 0.0002, *P*_PRL–PL_ = 0.0002, *P*_PRL–ML_ = 0.0005, *P*_PRL–SCL_ = 0.0003, ALT: *P*_PRL–normal_ = 0.9835, *P*_PRL–untreated_ < 0.0001, *P*_PRL–PL_ < 0.0001, *P*_PRL–ML_ < 0.0001, *P*_PRL–SCL_ < 0.0001). Results are presented as mean ± S.D. CDAHFD, a choline-deficient, L-amino acid-defined high-fat diet; HSC hepatic stellate cell; UT untreated group. PL plain PEGylated liposomes; ML maleimide-activated PEGylated liposomes; SCL scrambled cys-promelittin peptide-tagged liposomes; PRL promelittin-modified liposomes; CD26 cluster of differentiation 26; CD31 cluster of differentiation 31; Ck7 cytokeratin7; αSMA alpha-smooth muscle actin; ALT alanine transaminase; AST aspartate transaminase. Source data are provided as a Source Data file.

The in vivo efficacy of PRL was evaluated in the choline-deficient, L-amino acid-defined high-fat diet (CDAHFD)-induced model. The liposome treatment scheme for CDAHFD-induced model mice is illustrated in Fig. 7a. Compared to normal liver tissues, those of CDAHFD-induced model mice showed upregulated expression of FAP on αSMA-positive aHSC (Fig. 7b). FACS (Fig. 7c) and flow cytometric analysis (Fig. 7d) revealed that the level of FAP-positive cells was 5.2-fold higher in the CDAHFD-induced model compared to normal liver.

Histological evaluation with Sirius red, Masson’s trichrome, and H&E staining consistently revealed that PRL had an antifibrotic effect. In Sirius red staining, the fibrotic regions were significantly smaller in the group treated with PRL compared to the other groups (Fig. 7e, f). Image analysis of Sirius red staining showed that the fibrotic region was 3.2-fold smaller in the PRL-treated group compared to PL-treated group. Masson’s trichrome staining showed that the fibrotic region was lowest in the liver tissue of PRL-treated mice (Fig. 7g, h). Hydroxyproline levels in PRL-treated mice were 3.1-fold lower than those in untreated mice (Fig. 7i). The serum levels of AST (Fig. 7j) and ALT (Fig. 7k) were lowest in the group treated with PRL among the tested groups.

## Discussion

In this study, we demonstrated that promelittin on PRL can be specifically cleaved by FAP in fibrotic liver tissues. The FAP-liberated melittin was shown to be capable of killing aHSC and alleviating fibrosis in mouse models of liver fibrosis induced by BDL, CCl_4_, or CDAHFD. The cleavage of PRL in liver tissues was visualized by molecular imaging of FQL. In the liver fibrosis model, PRL treatment exerted an antifibrotic effect and especially prolonged survival in BDL mice.

Recently, various strategies have been reported to induce antifibrotic effects. For example, hepatic macrophages have been targeted in relaxin-mediated liver fibrosis therapy. Relaxin is known to bind to aHSC and hepatic macrophages, and the binding of relaxin to macrophages was very recently reported to switch them from the profibrogenic to pro-resolution phenotype^13^. Other strategies have sought to induce antifibrotic effects by metabolic regulation aimed at inhibiting the activation of HSCs. For example, an adiponectin-based agonist was shown to regulate lipid and glucose metabolism through binding to relevant receptors, and thereby suppress HSC activation^14^. Cotadutide, which is an agonist of glucagon receptor and glucagon-like protein-1 receptor, was reported to alleviate fibrosis via modulating lipogenesis in hepatocytes^15^.

In this study, different from inhibiting the activation of HSCs, PRL can selectively kill FAP-expressing aHSC to exert antifibrotic effects. The fibrosis-stimulated antifibrotic effect of PRL may prove useful for treating fibrosis in various fibrosis models. Indeed, we herein show that PRL exhibited antifibrotic effects in animal models induced by BDL surgery, CCl_4_, or high-fat diet. Our results further show that PRL selectively kills aHSC, not macrophages, suggesting that the combination of PRL with relaxin gene therapy may widen the spectrum of effective antifibrotic strategies by inhibiting the activation of HSCs and depleting aHSC (Supplementary Fig. 1).

In this study, we observed that PRL has substantial antifibrotic effects in the fibrosis models induced by CCl_4_ or high-fat diet. The severity of fibrosis reportedly depends on the dose and duration of CCl_4_ injection or high-fat diet feeding^16^. The dosing regimens of antifibrotic agents have differed across various studies, and different regimens have yielded different outcomes. For example, when adiponectin agonist treatment was initiated at week 3 of CCl_4_ injection, liver fibrosis was still evident 3 weeks later^14^. In another study, relaxin-based gene therapy was initiated at week 4 of CCl_4_ injection or week 8 of high-fat diet feeding, and complete absence of fibrosis was observed at 2 weeks after the initiation of gene therapy in both models^13^. When an antagonist of the binding of arginine-glycine-aspartate to integrin was delivered at week 6 of high-fat diet feeding, liver fibrosis was significantly ameliorated at week 4 after the initiation of antagonist treatment^17^.

To obtain an optimal antifibrotic effect, we used a dosing regimen wherein PRL injection was initiated at week 6 of CCl_4_ injection or week 10 of high-fat diet feeding. Compared to studies in which treatment began at weeks 3 or 4 of CCl_4_ injection or weeks 6 to 8 of high-fat diet feeding, we began injecting PRL at later phases of CCl_4_ or nonalcoholic steatohepatitis (NASH) model development. FAP expression intensity has been shown to correlate with the severity of liver fibrosis^8,18–20^. Since PRL needs FAP-expressing aHSC to exhibit antifibrotic effects, we hypothesized that progressed fibrosis would activate the antifibrotic effect of PRL more effectively than early-phase fibrosis. Indeed, we observed that the antifibrotic effect of PRL was less intense when PRL was given at week 4 of CCl_4_ treatment compared to the utilized regimen of starting at week 6 of CCl_4_ induction (Supplementary Fig. 7). The increased effect of PRL, when given at the later phase of fibrosis, seems to support the fibrosis-activated antifibrotic effect of PRL.

FAP-specific activation of PRL would be crucial for focusing the effects of melittin on target fibrotic cells and minimizing possible side effects of melittin in the bloodstream. To prevent the action of melittin on blood cells, we conjugated inactive promelittin to the surface of ML. Cys-promelittin was covalently tethered to the surface of ML using the maleimide reaction. Because a sulfide group is required for the maleimide reaction, we used cys-promelittin, in which a cysteine amino acid residue was added to the N-terminus of promelittin. RBC hemolysis assays support the conclusion that PRL are inactive against RBCs.

Instead of using the promelittin peptide per se, we used liposome formulations containing promelittin on the surface. PRL-bound promelittin has several potential advantages over free promelittin peptide. First, intravenous injection of nanoparticles has been reported to provide the highest distribution to the liver^21^. This is consistent with other previous reports showing that intravenously injected liposomes^22^, polymeric micelles^23^, and other nanoparticles^24^ are distributed to the liver to a greater extent than to other organs. Second, the tethering of promelittin on the PEGylated surface of PRL may prevent the nonspecific adsorption of proteases to liposomes. PEGylation has been reported to reduce the nonspecific opsonization and adsorption of proteins onto liposomes^25^. Here, the use of PEGylated liposomes for PRL could contribute to reducing nonspecific interactions with and cleavage of promelittin peptide by proteases in the blood. Third, the use of PRL provides the further opportunity to deliver both melittin and other antifibrotic chemical drugs, such as silibinin^26,27^ and quercetin^28^. Although we used PRL without chemical agents to demonstrate proof-of-concept in this study, the co-delivery of other antifibrotic chemical drugs loaded inside liposomes could further increase the antifibrotic efficacy of PRL.

Quenching and dequenching FRET phenomena have been used to test the cleavage of substrate peptides by enzymes in vivo. For example, a capsid protease-sensitive FRET system has been used as a proteolytic assay for the chikungunya virus^29^, and a FRET-based platform employing fluorescent carbon dots and MnO_2_ nanosheets have been used to detect glutathione in human blood^30^. Here, we tested the FAP-specific cleavage of promelittin by assessing FAP-mediated recovery of fluorescence of FQL, composed of promelittin peptide dually modified with the fluorophore EDANS and quencher DABCYL. In the absence of FAP, FQL remain quenched owing to FRET between the fluorophore and the quencher. However, the cleavage of promelittin peptide by FAP abrogates the close interaction of EDANS with the quencher, resulting in the recovery of EDANS fluorescence. We found that not only was FQL fluorescence recovered by FAP in a cell-free system, it was also recovered by FAP-positive LX-2 cells, but not by Chang cells. Importantly, fluorescence was also increased in the livers of BDL mice intravenously injected with FQL, confirming cleavage of promelittin peptide in vivo. The recovery of FQL fluorescence both in vitro and in vivo supports the cleavage of promelittin on PRL by FAP in fibrotic liver tissues. The specific cleavage of PRL by FAP on fibrotic liver cells may enhance the activity of liberated melittin towards FAP-positive cells.

In this study, we observed that the treatment of activated LX2 cells with siFAP significantly reduced the expression of FAP without affecting cell viability under PRL treatment. This might occur because the cytotoxic effect of melittin is nonlinear (Supplementary Fig. 5): The viability of activated LX-2 cells treated with 1.0 µg/ml melittin was 100%, whereas this viability decreased to 20% following treatment with 2.0 µg/ml melittin. To exert notable cytotoxicity against LX2 cells, therefore, melittin must be liberated at a level greater than its minimal effective concentration.

Our molecular imaging studies revealed that the activation of PRL was specific to the liver of BDL model mice, and was not seen in other organs. The notable fluorescence intensities of FQL at the liver of BDL model mice supports the notion that the fluorescent signal is selectively liberated by the increased FAP expression in liver tissues of BDL model mice. We herein measured the activation of FQL using ex vivo organ tissues rather than in vivo whole-animal imaging because the fluorescence signal liberated from FQL has an excitation wavelength of 335 nm, which limits in vivo imaging. The results of our ex vivo imaging suggest that there was little FAP-mediated activation of PRL in other vital organs, such as the heart, lung, spleen, and kidney. This lack of activation in other vital organs indicates that there is little possibility that pore-forming melittin will be liberated at nontarget organs.

The antifibrotic efficacy of PRL is attributable to reductions in the population of aHSC through the cytotoxic action of liberated melittin. FAP was observed to be overexpressed in stellate cells from the fibrotic liver, but not those from normal liver. Hepatic stellate cells in the fibrotic liver are known to be abnormally activated and overproduce collagen fibers^31^. The reduction of abnormally high aHSC populations may decrease the production of collagen fibers, and thereby reduce fibrosis. In this study, we observed that PRL, which killed FAP-positive sHSC, reduced the levels of hydroxyproline, a metabolite of collagen fibers^32^. The decrease in collagen fibers in the BDL liver also supports the efficacy of PRL against collagen fiber formation. We observed that PRL did not affect the population of FAP-negative cells such as macrophages, hepatocytes, and endothelial cells. The decrease of cholangiocytes by PRL treatment might be attributed to the amelioration of fibrosis. It has been reported that cholangiocytes are involved in the liver fibrogenesis^33^.

Although we demonstrated the antifibrotic efficacy of PRL in a liver fibrosis model, the selective activation of PRL by FAP suggests the potential of PRL for the treatment of various fibrotic diseases in which FAP is overexpressed. FAP has drawn considerable attention as a target enzyme in various fibrotic diseases and cancer. In the tumor microenvironment, cancer-associated fibroblasts have been reported to overexpress FAP^34^. In addition to cancers, fibrotic livers and inflamed areas^9^, as well as regions of lung fibrosis^35^, have been shown to overexpress FAP. Moreover, although we used promelittin to demonstrate FAP-specific activation in fibrotic tissues, our strategy could be applied to design other FAP-specific activation systems for fibrotic tissues. For example, other bioactive antifibrotic peptides can be converted to a form that is inactive in the bloodstream by introducing additional sequences and tethering the peptides to the surface of liposomes. Accordingly, FAP-selective cleavage in fibrotic tissues can confer targeted therapeutic efficacy of antifibrotic peptides in pathogenic sites.

In this study, we observed 100% mortality at 10 days after BDL surgery in BALB/c mice. It has been reported that the mortality of mice after BDL surgery can be affected by the utilized mouse species: Compared to C57BL/6 mice, BALB/c mice are more sensitive to liver fibrosis and show lower survival after BDL surgery^36^. The techniques used for BDL surgery are also expected to impact the mortality rate. BDL surgery can be done using either single-knot ligation or double-knot ligation with resection^37^. Single-knot ligation decreases but does not completely block the flow of bile through the bile duct, reflecting the conditions of cholestasis. Double-knot ligation with resection, which is often used to establish acute liber fibrosis, blocks bile flow and is associated with lower survival than single-knot ligation. The high mortality rate seen in our mouse model might be thus attributed to the use of the double-knot ligation method for BDL surgery in more susceptible BALB/c mice.

In conclusion, we showed that PRL is selectively activated by FAP overexpressed in fibrotic liver and effectively alleviate fibrosis. The FAP-specific liberation of melittin in the fibrotic liver can reduce the population of abnormal hepatic stellate cells and reduce the overproduction of collagen fibers. PRL encapsulating other antifibrotic chemical agents in their interior may be designed to increase antifibrotic efficacy. Notably, the PRL described here also provides insight into the design of systems with FAP-selective activation of other nanomaterials for effective treatment of a range of fibrotic diseases in which FAP is known to be overexpressed.

## Methods

### Animals

All animals (*n* = 5 biologically independent mice per group) were maintained and used in accordance with Guidelines for the Care and Use of Laboratory Animals of the Institute of Laboratory Animal Resources, Institutional Animal Care and Use Committee of Seoul National University (Seoul, Republic of Korea; approved animal experimental protocol number, SNU-130129-3-1). Eight-week-old female BALB/c mice and female C57BL/6 mice (Raon Bio Co., Gyeonggi-do, Republic of Korea) were used for in vivo experiments. All mice were housed up to 5 per cage with a 12 h light/dark cycle allowed ad libitum access to food (Rodent Chow; Cat# 38057, Purina Lab, Missouri, USA) and water under the ambient temperature of 23 ± 2 °C and humidity of 50 ± 10%. We have complied with all relevant ethical regulations for animal testing and research.

### Preparation of promelittin peptide-modified liposomes

Liposomes surfaces were modified with promelittin peptide by covalently tethering the Cys-promelittin peptide, N-CEPEAEADAEAGPAGIGAVLKVLTTGLPALISWIKRKRQQ-C, via a maleimide reaction. Liposomes were prepared by mixing egg L-α-phosphatidylcholine (PC; Avanti Polar Lipids, Alabaster, AL, USA), egg L-α-phosphatidyl DL-glycerol (PG; Avanti Polar Lipids), cholesterol (Chol; Sigma-Aldrich), and 1,2-distearoyl-sn-glycero-3-phosphoethanolamine-N-[maleimide(polyethylene glycol)-2000] (mal-PEG-DSPE; Avanti Polar Lipids) at a molar ratio of 2:2:2:0.4 in chloroform. For comparison, 1,2-distearoyl-sn-glycero-3-phosphoethanolamine-N-[(polyethylene glycol)-2000] (PEG-DSPE; Avanti Polar Lipids) was used in place of mal-PEG-DSPE in some experiments. After removal of chloroform using a rotary evaporator, the resulting thin lipid films were hydrated with 1 mL of phosphate-buffered saline (PBS; pH 7.4), vortexed, and extruded three times through 0.2 μm polycarbonate membrane filters (Millipore Corp., Billerica, MA, USA). This process resulted ML or PL. The resulting ML were further used to prepare PRL. For preparation of PRL, 0.8 μmol cys-promelittin (Peptron, Daejeon, Republic of Korea) was mixed with 1 mL of ML containing 0.4 μmol of mal-PEG-DSPE. The cysteine in the peptide was reacted with maleimide groups of ML by incubating at room temperature for 6 h.

In some experiments, a scrambled cys-promelittin sequence (N-CEAGAEPAA EPKPATSGDILWVLAARLTVLIGEQKQKRIG-C) or a promelittin-mimicking fluorescence-quenched peptide [CEPEAEADA-E (fluorophore)-AGPAGIGAVLK-quencher], was linked to the surface of ML, resulting in SCL and FQL, respectively. This latter dual-modified promelittin-mimicking peptide, containing 5-[(2-aminoethyl) amino]naphthalene-1-sulfonic acid (EDANS) as a fluorophore and 4-((4-(dimethylamino) phenyl)azo)benzoic acid (DABCYL) as a quencher, was prepared as illustrated in Fig. 2g. The EDANS-DABCYL–linked promelittin-mimicking peptide was tethered to the surface of ML containing 0.4 μmol of mal-PEG-DSPE. The resulting FQL was purified from unreacted peptides by chromatography on a PD-10 column (GE Healthcare Life Science, Cat. No. 17-0851-01, Mickleton, NJ, USA) and stored at 4 °C until use. FAP-sensitive cleavage of promelittin peptide on liposomes was tested by monitoring fluorescence recovery of the quenched fluorophore.

### Quantification of peptides on liposomes

The amount of peptides on liposomes was assayed using fluorescamine, a heterocyclic dione that reacts with primary amines of peptides^38^. Specifically, an aliquot (32 μL) of fluorescamine solution (Sigma, cat. No. F-9015; 3 mg/mL in acetonitrile) was added to 100 μL of peptide-conjugated liposomes, and the mixture was allowed to react at room temperature for 10 min. The fluorescence intensity of fluorescamine-labeled liposomes was assessed at an excitation wavelength of 365 nm and an emission wavelength of 470 nm using a SpectraMAX M5 Multi-Mode Microplate Reader (Molecular Devices, San Jose, CA, USA). For calculation of peptides on liposomes, a standard curve was generated using serial dilutions of a cys-promelittin standard solution. The amount of phospholipids in liposomes was determined using a phosphate assay as previously described^39^.

### Characterization studies

Liposomes were characterized by size, zeta potential, and morphology. The size and zeta potential of various liposome preparations were measured using dynamic light scattering and laser-Doppler micro-electrophoresis, respectively. The hydrodynamic diameters of liposomes were measured at an angle of 90° at 24.1 °C using a HeNe laser (10 mW) and an ELSZ-1000 dynamic light-scattering instrument (Photal Otsuka Electronics Co., Osaka, Japan). Zeta potential was measured at an angle of 22°. Data were analyzed using the ELSZ-1000 software (ver. 5.10) package supplied by the manufacturer. TEM images were acquired using a Talos L120C TEM system (FEI, Brno, Czech) operating at 120 kV. Liposomes were loaded onto a 300-mesh copper grid coated with formvar-carbon (01753-F; Ted Pella, Redding, CA, USA). The grid was washed twice with distilled water and then negative-stained with 1% (w/v) uranyl acetate, after which images of negatively stained liposomes were obtained.

### Stability test

The stability of liposomes was monitored up to 18 days in the presence and absence of FAP (5 nM). For 18 days, liposomes were stored with or without FAP at 4 °C or room temperature. The hydrodynamic diameters of liposomes were measured 3 day intervals using an ELSZ-1000 (ver.5.10) instrument (Photal Otsuka Electronics Co.).

### FAP-mediated fluorescence recovery test

The cleavage activity of FAP towards promelittin sequences was tested using the quenching and dequenching feature of the promelittin-mimicking fluorescence-quenched peptide on FQL. FQL (0.9 mg phospholipid/mL) were incubated with 1 μM FAP or matrix metalloproteinase 9 (MMP9; R&D Systems Inc.) at 37 °C for 1 h. Recovery of the fluorescence of EDANS liberated from the nearby quencher DABCYL was assessed by fluorometry at an excitation wavelength of 335 nm and an emission wavelength of 490 nm using a SpectraMAX M5 system (Molecular Devices).

### Hemolysis assay

The hemolysis activity of various liposomes was tested using mouse RBCs. RBCs (2 × 10^4^ cells/mL) were isolated from BALB/c mice and incubated with various liposome preparations in the absence or presence of 1 μM FAP. RBCs treated with 1% Triton X-100 served as a 100% hemolysis positive control, and a suspension of RBCs in PBS was used as a negative control. After centrifuging samples at 1000 × *g* for 10 min, supernatants containing hemoglobin released from lysed RBCs were transferred to a clear, flat-bottomed, 96-well polystyrene plate (SPL Life Sciences, Pocheon, Republic of Korea). The percentage of hemolysis was calculated by measuring the absorbance of each sample at 540 nm using a SpectraMax Plus plate reader (Molecular Devices).

### Release kinetics of PRL

To evaluate the release kinetics of melittin by FAP, PRL (0.9 mg phospholipid/mL) was incubated without or with 5 nM of FAP. At various time points, the released melittin was separated from PRL by filtration with a 50-kDa membrane filter (Millipore, Billerica, MA, USA). The amounts of released melittin were measured using a fluorescamine assay^38^.

### FAP-mediated release kinetics of DABCYL-modified peptides from FQL

To evaluate the FAP-mediated release kinetics of fluorescent melittin from FQL, we incubated FQL (0.9 mg phospholipid/mL) without or with 5 nM of FAP or matrix metalloproteinase 9 (MMP9; R&D Systems, Inc.) at 37 °C for various periods. The recovery of fluorescence representing EDANS-labeled melittin peptide liberated from FQL was assessed by fluorometry at an excitation wavelength of 335 nm and an emission wavelength of 490 nm using a SpectraMAX M5 system (Molecular Devices).

### Cell culture

LX-2 human hepatic stellate cells (#SCC064, Sigma-Aldrich, kindly provided by Professor Sang Geon Kim, College of Pharmacy, Dongguk University, Republic of Korea) and FAP-negative Chang cells (ATCC CCL-13; kindly provided by Professor Mi-Ock Lee, College of Pharmacy and Research Institute of Pharmaceutical Sciences, Seoul National University, Republic of Korea) were used for testing FAP-mediated cleavage of peptides on various liposome preparations. Chang cells are listed in the ICLAC register as a misidentified cell line. Cells were cultured in Dulbecco’s modified Eagle medium (DMEM) supplemented with 10% fetal bovine serum (FBS), 100 units/mL of penicillin, and 100 μg/mL of streptomycin at 37 °C in a humidified 5% CO_2_ atmosphere. For activation, LX-2 cells were stimulated with TGF-β1 (transforming growth factor-β1) (10 ng/ml; GenScript Biotechnology, Nanjing, China) for 24 h. In some experiments, LX-2 cells treated with or without small interfering RNA (siRNA) targeting FAP (siFAP) were seeded onto six-well plates (SPL Life Sciences, Pocheon, Republic of Korea) at a density of 4 × 10^5^ cells/well and then incubated for 24 h to ~80% confluence.

### Assessment of siFAP efficiency

The efficiency of siFAP was assessed by measuring the expression levels of FAP using flow cytometry. LX-2 cells were cultured in DMEM supplemented with 10% FBS, 100 units/mL of penicillin, and 100 μg/mL of streptomycin. LX-2 cells (4 × 10^5^ cells/well) were seeded to a six-well plate (SPL Life Sciences), incubated for 24 h, and transfected for 20 min with 50 nM siFAP (Bioneer Corporation, Daejeon, Republic of Korea) complexed with 5 μL of Lipofectamine 2000. The sequences of siFAP were 5′-CUC UAU GCA GUG UAU CGA AdTdT-3′ (sense) and 5′-UUC GAU ACA CUG CAU AGA gdTdT-3′ (antisense). In some experiments, scrambled-sequence siRNA (siSCR) was used as a control. After transfection, cells were incubated for an additional 48 h. Next, cells were stained with a rabbit anti-FAP primary IgG antibody (1:50, Abcam) for 1 h, followed by an allophycocyanin (APC)-conjugated goat anti-rabbit IgG antibody (1:100, Abcam). After staining, cells were analyzed via BD LSR Fortessa (BD Bioscience) and Leica TCS8 confocal microscope (Leica, Bensheim, Germany). Flow cytometry data were acquired with BD FACSDIVA™ (v8.0.1., BD Bioscience). LEICA Application Suite X (v3.6.0., Leica) was used to collect confocal images. Control cells were stained with rabbit IgG isotype antibody (1:50, Abcam) followed by an APC-conjugated goat anti-rabbit IgG antibody (1:100, Abcam).

### MTT assay

Cell viability after treatment with various liposome preparations was assessed by MTT [3-(4,5-dimethylthiazol-2-yl)-2,5-diphenyltetrazolium bromide] assay. Briefly, LX-2 cells were seeded onto 24-well plates at a density of 6 × 10^4^ cells/well. After cells reached 70% confluence, they were treated with various liposome preparations for 24 h. MTT solution (500 μM) was then added to each well and plates were incubated for 2 h. The medium was then aspirated and 200 μL of dimethyl sulfoxide (DMSO; Sigma-Aldrich) was added to dissolve formazan crystals generated by metabolically active (live) cells. The viability of cells was quantified by measuring optical density at 570 nm using a microplate reader (Tecan Group Ltd., Seestrasse, Mannedorf, Switzerland).

### Establishment of the bile duct-ligation model

Liver fibrosis was induced in mice by performing BDL surgery under anesthesia (isoflurane 1.5%)^40^ with slight modifications. The surgical area was shaved and cleaned preoperatively using a povidone-iodine solution, then an incision was made in the upper midline and the bile duct was exposed using a wet swab. The bile duct was ligated with silk thread knots at two sites and then cut between the knots, after which the abdominal wall was closed using nonabsorbable silk thread. On days 1 and 3 after BDL surgery, mice were intravenously injected with various liposome preparations.

### In vivo efficacy test in carbon tetrachloride induced liver fibrosis model

To test the in vivo antifibrotic effect of PRL, the CCl_4_-induced chronic liver fibrosis model was established. Eight-week-old female C57BL/6 mice were intraperitoneally injected with CCl_4_ 1 ml/kg (25% CCl_4_ in olive oil) twice a week for 6 weeks^16^. The liver fibrosis-bearing mice were then intravenously treated with the various liposomes every other day for 2 weeks and sacrificed at 2 weeks after the first treatment. Blood and liver tissues were collected for further analysis. To test the in vivo antifibrotic effect of PRL in an earlier phase of the CCl_4_-induced liver fibrosis model, 8-week-old female C57BL/6 mice were intraperitoneally injected with CCl_4_ at 1 ml/kg (25% CCl_4_ in olive oil) twice a week for 4 weeks. The liver fibrosis-bearing mice were then intravenously treated with PRL every other day for 2 weeks and sacrificed at 4 weeks after the first PRL treatment. Liver tissues were collected for analysis.

### In vivo efficacy test in high fat diet-induced liver fibrosis model

The high fat diet-induced liver fibrosis model was established^41^. Mice were fed with CDAHFD (Cat#A06071302; Research Diets, New Brunswick, NJ) for 10 weeks. For normal control group, mice were fed with chow diet (Rodent Chow; Cat# 38057, Purina Lab, Missouri, USA). The mice were then treated with the various liposomes (dose = 0.27 mg phospholipid/mouse) every other day for 2 weeks and sacrificed 1 day after the last treatment. Blood and liver tissues were collected for further analysis.

### FAP expression analysis by flow cytometry

FAP expression on aHSC of liver tissues was analyzed via flow cytometry. Single-cell suspensions of liver tissue were obtained as previously described with slight modification treatment^42^. Briefly, liver tissues were perfused with HEPES buffer (Sigma-Aldrich; cat. No. H4034) containing collagenase D (Sigma-Aldrich; cat. No. C5138-1G) and pronase (Sigma-Aldrich), and digested with stirring at 37 °C for 30 min. Cells were collected by centrifugation at 580 × *g* for 10 min, and dead cells were excluded using a Zombie Red Fixable Viability Kit (BioLegend). The cells were then stained with PE/Cyanine7-conjugated rat anti-mouse CD26 antibody (1:100, BioLegend; cat. No. 137810), PE-conjugated rat anti-mouse CD31 antibody (1:100, BioLegend; cat. No. 102407, Lot. No. B261070), PerCP/Cy5.5-conjugated rat anti-mouse CD45 antibody (1:50, BioLegend; cat. No. 103132, Lot. No. B282872), and rabbit anti-mouse FAP antibody (1:50, Abcam; cat. No. ab28244, Lot No. GR217381-54) for 1 h at 4 °C. Secondary antibody staining was performed with Alexa Fluor 647-conjugated goat anti-rabbit IgG antibody (1:200, Abcam; cat. No. ab150083, Lot No. GR3370563-1) for 30 min at 4 °C. Cells were analyzed via BD LSR Fortessa (BD Bioscience). Flow cytometry data were acquired with BD FACSDIVA™ (v8.0.1., BD Bioscience). The HSC population was gated as CD26-CD31-CD45-VitA+ cells using FACS; quadrants for FAP + HSC were selected relative to the fluorescence minus one (FMO) control.

### Molecular imaging

The organ distribution of FQL was determined by molecular imaging. Five days after BDL surgery, BALB/c mice (5 mice per group) were intravenously injected with FQL at a dose of 0.27 mg phospholipid/mouse. One hour later, vital organs (i.e., liver, heart, lung, spleen, and kidney) were isolated and their fluorescence intensities were assessed using an In Vivo Imaging System (IVIS; PerkinElmer, Hopkinton, MA, USA) (*n* = 5 independent samples per group). Living image (v4.5.2., PerkinElmer) was used to analyze fluorescent images of organs.

### Assay of collagen in liver tissue

The amount of collagen in liver tissues was determined by measuring the content of hydroxyproline using a Hydroxyproline assay kit (Abcam, Cambridge, UK) as described by the manufacturer. Briefly, 4 days after BDL surgery, mice treated with various formulations were sacrificed and their liver tissues were extracted and homogenized. The homogenate was then treated with 10 N NaOH, incubated at 120 °C for 1 h, and neutralized with 10 N HCl. After centrifugation at 10,000 × *g* for 5 min, the supernatant was added to wells of a 96-well plate and dried at room temperature. The resulting crystalline residue was dissolved with 0.1 mL of oxidation-buffered chloramine T solution and incubated at room temperature for 20 min. After addition of 50 μL of acidic developer solution, provided in the kit, the plate was incubated at 65 °C for 45 min and treated with 50 μL of the provided DMAB solution. Absorbance was measured at 540 nm using a SpectraMax Plus plate reader (Molecular Devices).

### In vivo assessment of liver function

The antifibrotic effects of liposomes were tested in vivo using a hematological parameter assay and histological staining. BDL surgery was performed on 8-week-old female BALB/C mice (Raon Bio). On days 1 and 3 after BDL surgery, mice were intravenously administered liposomes at a phospholipid dose of 2.7 mg/mL. Four days after BDL surgery, blood samples were collected and assayed for ALT, AST, bile acid, and total bilirubin levels by the Neodin VET Diagnostics Institute (Seoul, Republic of Korea). For histological assessment of liver tissues, the liver was extracted 4 days after BDL surgery, fixed in 10% formalin for 48 h, and paraffin-embedded. The tissues sections were analyzed with H&E staining, and Masson’s trichrome staining. For H&E staining, tissue sections were immersed in filtered Harris hematoxylin for 10 s and then in eosin for 30 s. The percentage of connective tissue areas in fibrotic regions on stained slides was calculated using a Vectra 3.0 Automated Quantitative Pathology Imaging System (PerkinElmer, Hopkinton, MA, USA). Images were analyzed using the InForm v2.4.11. software (Perkin-Elmer).

### Immunohistochemistry of liver tissues

FAP expression on aHSC in the liver was evaluated with immunofluorescent staining of formalin-fixed paraffin-embedded tissues. Liver tissues were sectioned at 4 µm thickness, de-paraffinized with xylene, and rehydrated with an ethanol series. Tissue slides were incubated with a Target Retrieval Solution (pH 6.0) (Agilent Dako, Santa Clara, CA, USA). After being washed, the slides were incubated with 0.1% Triton-X 100, blocked with 10% goat serum in PBS, and stained overnight at 4 °C with rabbit anti-mouse FAP antibody (1:100, Abcam; cat. No. ab28244, Lot No. GR217381-54) and mouse anti-mouse alpha-smooth muscle actin (αSMA) antibody (1:100, Abcam; cat. No. ab7817, Lot No. GR3356520-4). Alexa Fluor 594-conjugated goat anti-mouse IgG antibody (1:100, BioLegend; cat. No. 405326, Lot. No. B324994) and Alexa Fluor 647-conjugated goat anti-rabbit IgG antibody (1:200, Abcam; cat. No. ab150083, Lot No. GR3370563-1) were applied for 1 h at room temperature, and the slides were imaged using a Thunder imager 3D assay (Leica Microsystems GmbH, Wetzlar, Germany).

Populations of cells in the liver tissues were analyzed via immunohistochemistry using various cell markers. Fibrosis-induced mice were treated with various liposomes. One day after liposome treatment, mice were anesthetized using isoflurane and the liver was perfused via the vena cava with 30 mL of PBS, and then collected. Excised liver tissues were fixed in 4% paraformaldehyde for 48 h at 4 °C. After dehydration in 30% sucrose solution overnight, liver tissues were embedded in OCT compound (Sakura Finetek, Japan) and frozen at −80 °C. Frozen liver sections (8 μm thickness) were blocked with 10% goat serum (Abcam, Cambridge, UK) and stained with primary antibodies overnight at 4 °C. Alexa Fluor 594 rabbit anti-mouse alpha-smooth muscle actin (αSMA) antibody (1:100, Cell Signaling Technology, Danvers, MA, USA; cat. No. 36110S), phycoerythrin (PE)-conjugated rat anti-mouse F4/80 antibody (1:50, BioLegend, San Diego, CA, USA; cat. No. 123110), APC-conjugated rat anti-mouse CD26 antibody (1:100, BioLegend; cat. No. 137807), PE-conjugated rat anti-mouse CD31 antibody (1:50, Invitrogen, Waltham, MA, USA; cat. No. 12-0311-82), and rabbit anti-mouse cytokeratin 7 antibody (1:100, Abcam; cat. No. ab181598) with Alexa Fluor 647-conjugated goat anti-rabbit IgG antibody (1:200, Abcam; cat. No. ab150083, Lot No. GR3370563-1) were used as markers of HSCs, macrophages, hepatocytes, endothelial cells, and cholangiocytes, respectively.

After secondary antibody staining, tissues were counterstained with DAPI and examined by a VECTRA tissue analyzer (v3.0.5., Perkin-Elmer). Image analysis was applied using the InForm 2.2.1 image analysis software (Perkin-Elmer).

### qRT-PCR for analysis of cell types

Populations of cells in the liver tissues were also evaluated by running qRT-PCR. Total RNA was extracted from liver tissue using the TRIzol reagent (Invitrogen) and reverse-transcribed into cDNA by incubating at 42 °C for 60 min and 70 °C for 5 min using RT PreMix (Intron Biotechnology Inc., Seoul, Republic of Korea). The sequences of primers for qRT-PCR are listed in Supplementary Table 1. qRT-PCR was performed at Applied Biosystems 7500 Fast Real-Time PCR System (Thermo Fisher Scientific, Waltham, MA, USA) using TOPreal qPCR 2x premix (Enzynomics, Daejeon, Republic of Korea, cat. No. RT501M). The housekeeping gene, glyceraldehyde-3-phosphate dehydrogenase, was used for normalization of each mRNA expression level. Applied Biosystems^®^ 7300 Real-Time PCR System (v1.4.0.) was used to collect relative gene expression values.

### Terminal deoxynucleotidyl transferase dUTP nick end labeling (TUNEL) assay

To evaluate apoptosis of cells in liver tissues, the TUNEL assay was performed. In brief, frozen liver tissue sections (8 μm thickness) were stained with a DeadEnd™ Fluorometric TUNEL System (Promega) according to the manufacturer’s protocols. To identify the types of TUNEL-positive apoptotic cells, immunofluorescence staining was sequentially done using markers for various cells. Alexa 594-conjugated anti-αSMA antibody (1:100, Cell Signaling Technology, cat. No. 36110S), PE-conjugated anti-F4/80 antibody (1:50, BioLegend, cat. No. 123110), APC-conjugated anti-CD26 antibody (1:100, BioLegend; cat. No. 137807) and rabbit anti-mouse cytokeratin 7 antibody (1:100, Abcam; cat. No. ab181598) with Alexa Fluor 647-conjugated goat anti-rabbit IgG antibody (1:200, Abcam; cat. No. ab150083, Lot No. GR3370563-1) were used to mark aHSC, macrophages, and hepatocytes, respectively. After overnight incubation at 4 °C, DAPI staining and mounting were performed on the slides, and fluorescence images of slide were obtained by VECTRA (Perkin-Elmer). Then, images were analyzed by InForm 2.2.1 image analysis software (Perkin-Elmer) followed by quantification of co-localization of TUNEL signals with each cell marker.

### Safety study

To evaluate the toxicity of PRL, a survival study was conducted in normal mice. Eight-week-old BALB/c mice received repeated intravenous administrations of PL or PRL at a dose of 0.27 mg phospholipid/mouse. Mice were administered with PL or PRL four times over 2 weeks. The survival and body weight of mice were monitored over 60 days.

### Statistics

Experimental data were statistically analyzed by one-way analysis of variance (ANOVA) with Tukey test. All statistical analyses were performed using GraphPad Prism software (v8.0, GraphPad Software, San Diego, CA, USA). A *P*-value less than 0.05 was considered statistically significant.

### Reporting summary

Further information on research design is available in the Nature Research Reporting Summary linked to this article.

## Supplementary information

Supplementary Information

Description of Additional Supplementary Information

Reporting Summary

## Data Availability

All datasets generated in the study are included in the manuscript, the supplementary information, and source data. Source data for all figures are provided with this paper. Source data are provided with this paper.

## Source data

Source data

## Code availability

This study did not use any custom code.
