# Supplementary material for: Fibroblast activation protein activated antifibrotic peptide delivery attenuates fibrosis in mouse models of liver fibrosis
Source: Nat Commun. 2022 Mar 21;13:1516. doi: 10.1038/s41467-022-29186-8 (PMC8938482; doi:10.1038/s41467-022-29186-8)
Supplement: Supplementary file 1 — Supplementary Information [file 41467_2022_29186_MOESM1_ESM.pdf]

Supplementary information

Liver fibrosis-activated antifibrotic peptide delivery

Jaiwoo Lee[a,1], Junho Byun[a,1], Gayong Shim[b], Yu-Kyoung Oh[a,*]

[a]College of Pharmacy and Research Institute of Pharmaceutical Sciences, Seoul National University, 1 Gwanak-ro, Gwanak-gu, Seoul 08826, Republic of Korea

[b]School of Systems Biomedical Science, Soongsil University, Seoul 06978, Republic of Korea

* To whom correspondence should be addressed.

[1] These authors equally contributed to this work.

Tel: 82-2-880-2493; Fax: 82-2-882-2493

E-mail: ohyk@snu.ac.kr

- Supplementary Figure 1 – 7

- Supplementary Table 1

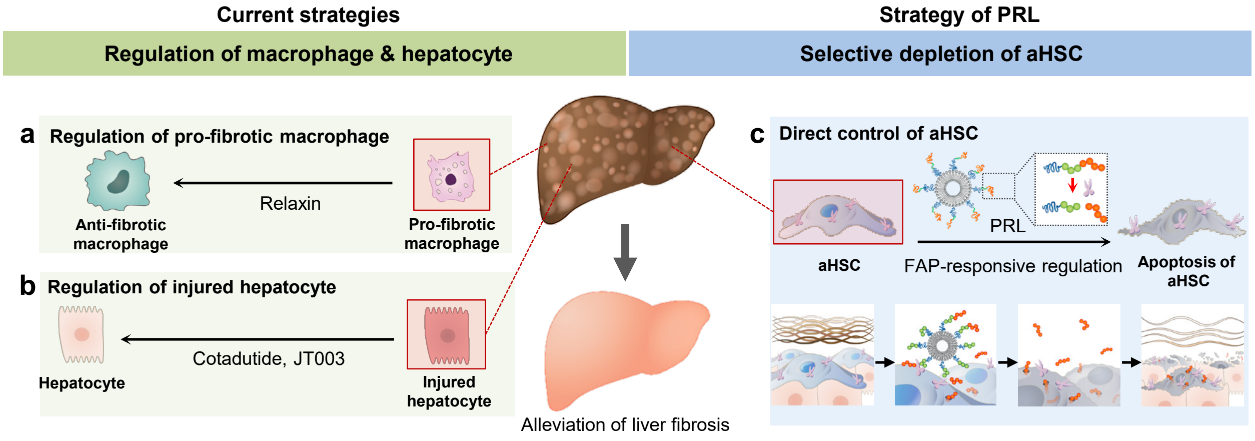

Supplementary Figure 1. Strategies for treatment of liver fibrosis. For alleviation of liver fibrosis, various strategies have been studied. (a) Pro-fibrotic macrophages were treated with anti-inflammatory peptides such as relaxin for switching to anti-fibrotic macrophages [13]. (b) Injured hepatocytes were treated with metabolism modulating agents such as adiponectin receptor agonists [14] or glucagon receptor agonists [15]. (c) In this study, aHSC was directly targeted with PRL via FAP-responsive activation of melittin. aHSC, activated hepatic stellate cells; FAP, fibroblast activation protein; PRL, promelittin-modified liposomes.

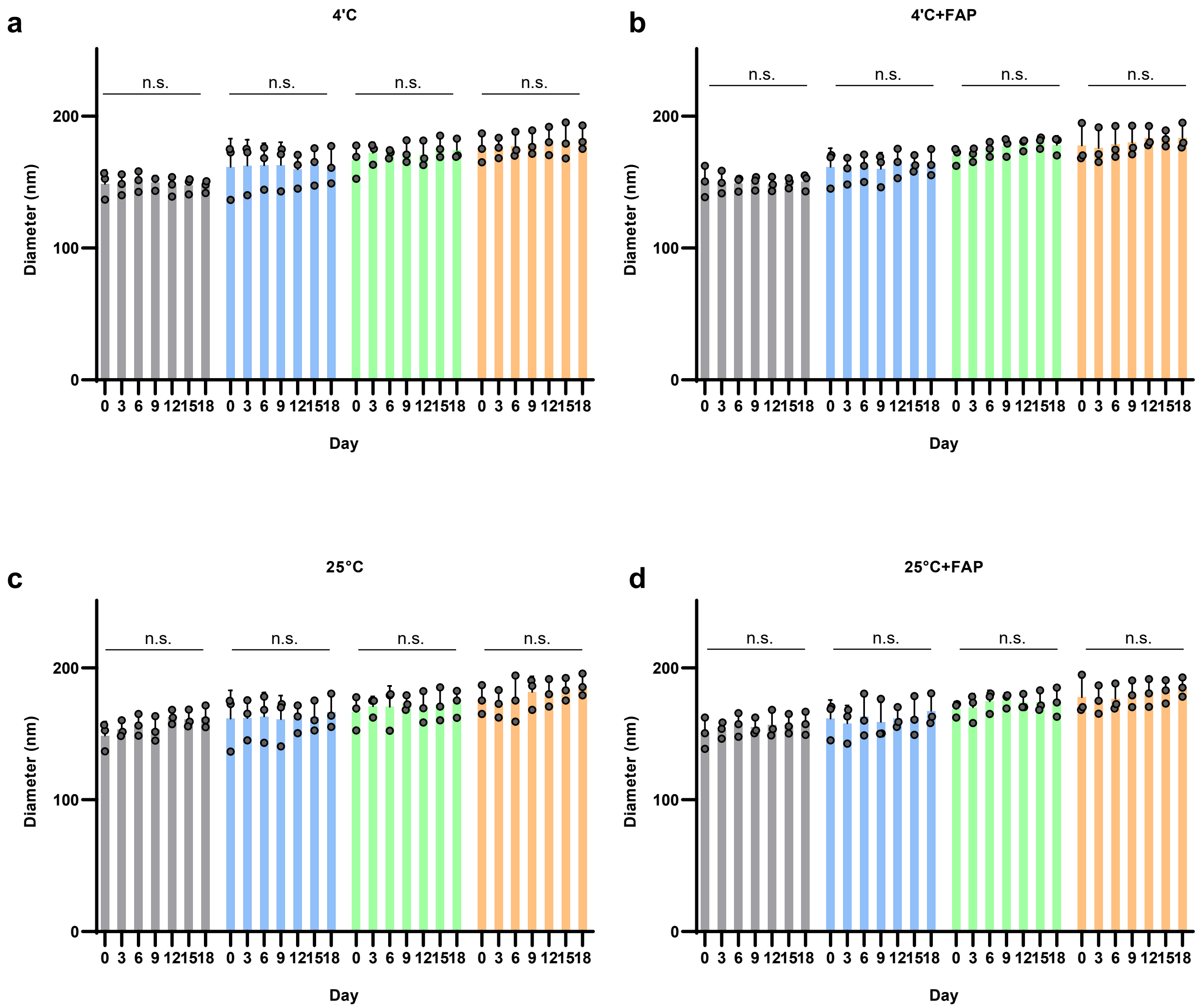

Supplementary Figure 2. Stability of PRL. Various liposomes (n=3 per group) were stored in the absence (a, c) or presence (b, d) of FAP over 18 days. As storage temperatures, 4°C (a, b), and 25°C (c, d) were used. The sizes of liposomes were measured by light scattering technique. n.s. :$P > 0.05$. All results are presented as mean ± S.D.. All significant differences were assessed using a one-way ANOVA with Tukey test (Tukey's test n.s. :$P > 0.05$). n.s.: not significant; FAP, fibroblast activation protein. Source data are provided as a Source Data file.

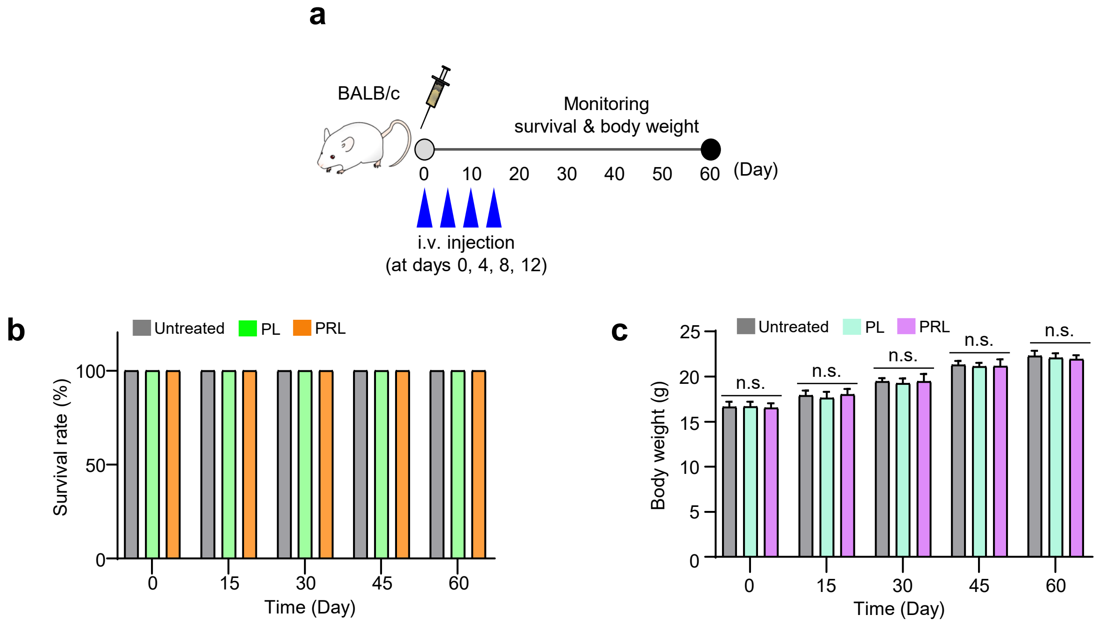

Supplementary Figure 3. Survival study of PRL. (a) Mice were untreated or treated four times with PL or PRL over 2 weeks. The survival rates were monitored over 60 days after the last dose. (b) Survival rates were shown for each group. (n = 5 mice per group). (c) Body weights of mice were shown for each group. (n = 5 mice per group). Significant differences were assessed using a one-way ANOVA with Tukey test (Tukey's test n.s. :P > 0.05). Results are presented as mean ± S.D.. n.s.: not significant; PL, plain PEGylated liposomes; PRL, promelittin-modified liposomes. Source data are provided as a Source Data file.

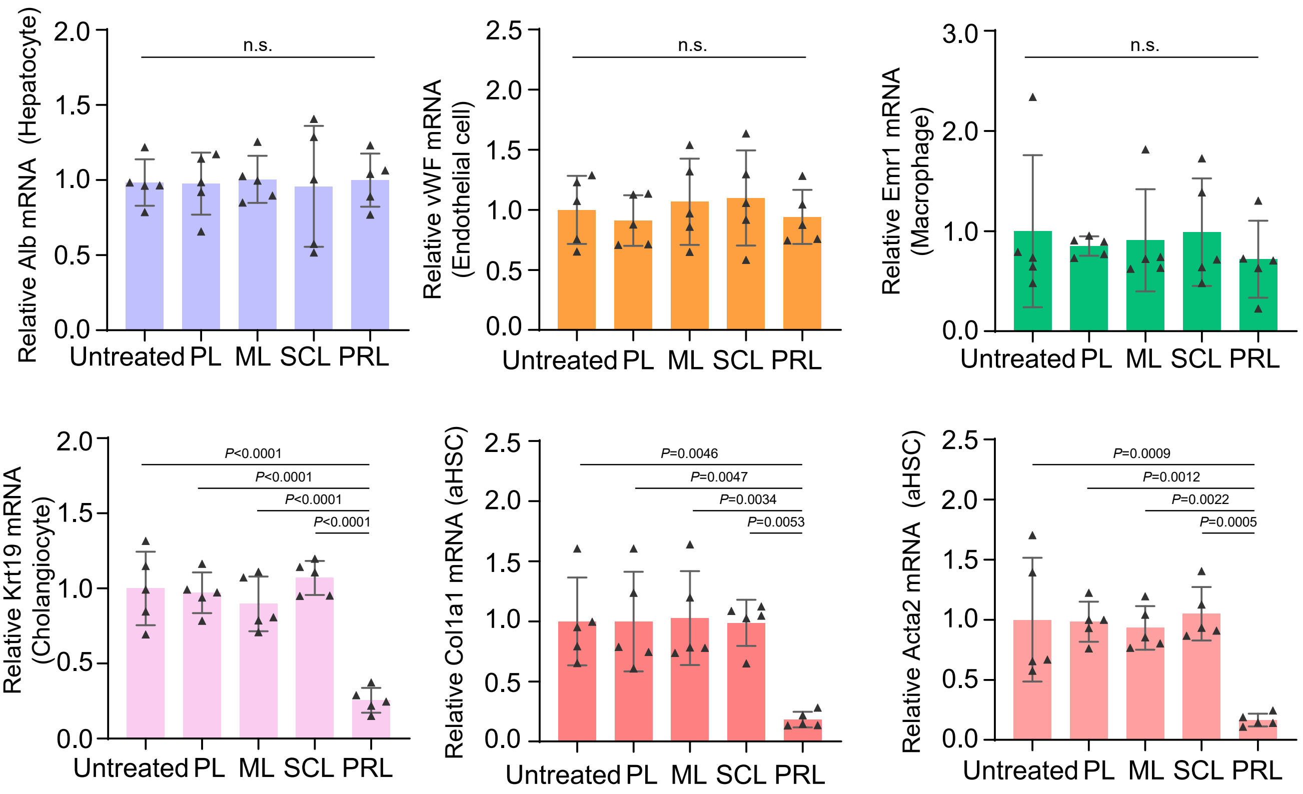

Supplementary Figure 4. Cell population analysis by qPCR in the liver tissue. BDL-induced mice were treated with various liposomes. Cell population in the liver tissue was evaluated using qRT-PCR. Total RNA was isolated form liver tissues and then reverse-transcribed into cDNA. qPCR was performed with the primers for Emr1 (macrophage), Alb (hepatocyte), vWF (endothelial cell), Krt19 (cholangiocyte) and Col1a1, Acta2 (aHSC). (n = 5 samples per group). All results are presented as mean ± S.D.. All significant differences were assessed using a one-way ANOVA with Tukey test (Tukey's test n.s. :P > 0.05, Krt19, $P_{PRL-untreated} < 0.0001$, $P_{PRL-PL} < 0.0001$, $P_{PRL-ML} < 0.0001$, $P_{PRL-SCL} < 0.0001$, Col1a1, $P_{PRL-untreated} = 0.0046$, $P_{PRL-PL} = 0.0047$, $P_{PRL-ML} = 0.0034$, $P_{PRL-SCL} = 0.0053$, Acta2, $P_{PRL-untreated} = 0.0009$, $P_{PRL-PL} = 0.0012$, $P_{PRL-ML} = 0.0022$, $P_{PRL-SCL} = 0.0005$). n.s.: not significant; PL, plain PEGylated liposomes; ML, maleimide-activated PEGylated liposomes; SCL, scrambled cys-promelittin peptide-tagged liposomes; PRL, promelittin-modified liposomes. Source data are provided as a Source Data file.

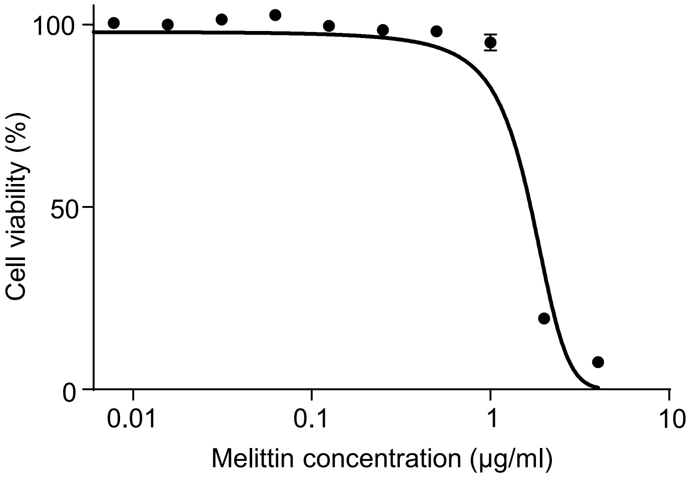

Supplementary Figure 5. Viability of cells treated with melittin. Activated LX-2 cells were treated with melittin peptides at various concentrations, and cell viability was measured by MTT assay. (n = 5 samples per group). All results are presented as mean ± S.D.. Source data are provided as a Source Data file.

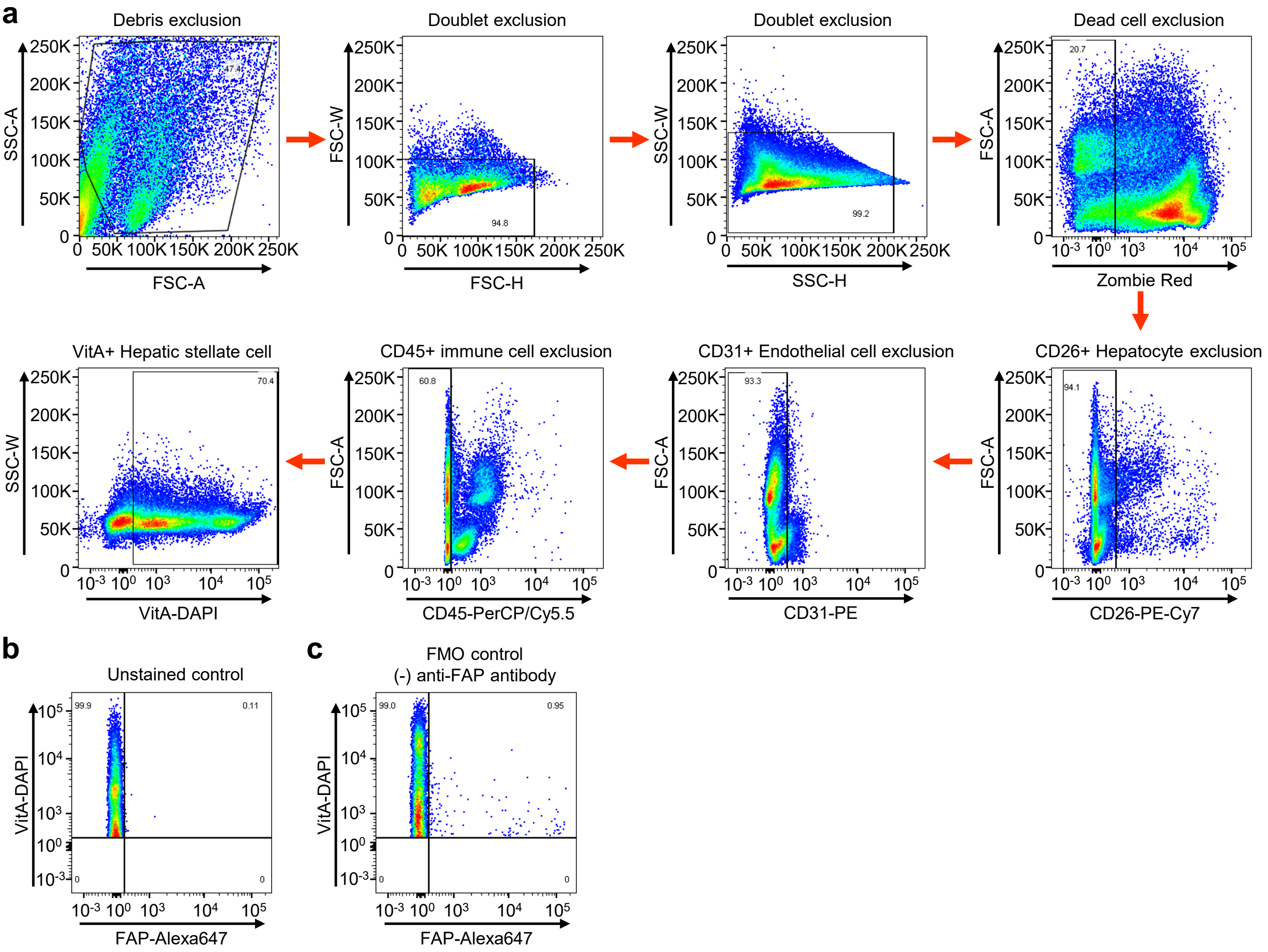

Supplementary Figure 6. Gating strategy and FMO control for FAP-positive aHSC. (a) FAP expression levels of the VitA+ cells plotted in Figures 4b, 6c, and 7c were gated from VitA+CD45-CD26-CD31- cells obtained from single-cell suspensions of liver. CD31, CD26, and CD45 were used as markers to exclude endothelial cells, hepatocytes, and immune cells, respectively. (b) Unstained control. (c) FMO control for FAP marker. FSC, forward scatter; SSC, side scatter; FAP, fibroblast activation protein; FMO, fluorescence minus one.

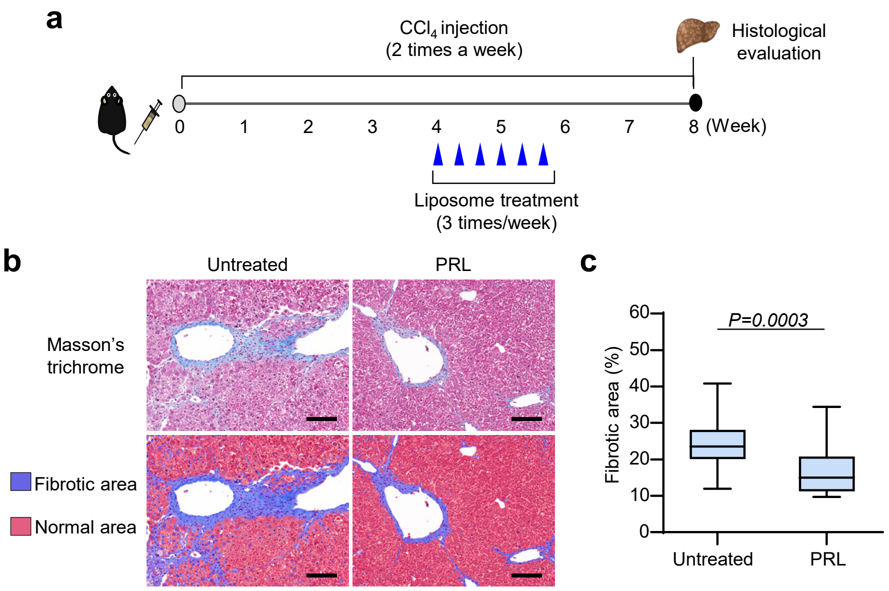

Supplementary Figure 7. In vivo antifibrotic effect of PRL treatment starting at week 4 of the CCl_4-induced liver fibrosis model. (a) Schematic of PRL dosing regimen. PRL treatment was initiated at week 4 of CCl_4 liver fibrosis model development and repeated thrice weekly for 2 weeks. (b) Representative Masson's trichrome staining in CCl_4-induced fibrotic liver tissues of untreated and PRL-treated groups. Fibrotic regions (blue) were distinguished from normal tissue (red) (n = 5 mice per group). Scale bar: 50 μm. (c) Fibrotic tissue areas were calculated from whole images of stained liver tissues. All significant differences were assessed using a one-way ANOVA with Tukey test (Tukey's test, $P_{PRL\text{–}untreated} = 0.0003$). In box plots, boxes show the 25th–75th percentile with the median, and whiskers show the minimum–maximum. PRL, promelittin-modified liposomes. Source data are provided as a Source Data file.

Supplementary Table 1. Primer sets for quantitative real time-PCR .

Cell type	Target gene	Primer sequence (5' – 3')	
		Forward	Reverse
HSC	Col1a1	CCTCAGGGTATTGCTGGACAAC	CAGAAGGACCTTGTTTGCCAGG
	Acta2	GCTGGTGATGATGCTCCCA	GCCCATTCCAACCATTACTCC
Hepatocyte	Alb	AGAAATCACCAAATTGGCAACA	TGCGCATTCCAGCAGGT
Endothelial cell	vWF	TGGATCCCGAGTCCTTTGTG	GAGTACAGGACATGCGCACTCT
Macrophage	Emr1	CCTGGACGAATCCTGTGAAG	GGTGGGACCACAGAGAGTTG
Cholangiocyte	Krt19	AATGGCGAGCTGGAGGTGAAGA	CTTGGAGTTGTCAATGGTGGCAC
