# Supplementary material for: Fibroblast activation protein activated antifibrotic peptide delivery attenuates fibrosis in mouse models of liver fibrosis
Source: Nat Commun. 2022 Mar 21;13:1516. doi: 10.1038/s41467-022-29186-8 (PMC8938482; doi:10.1038/s41467-022-29186-8)
Supplement: Supplementary file 2 — Description of Additional Supplementary Information [file 41467_2022_29186_MOESM2_ESM.pdf]

**Description of Additional Supplementary Information**

**Editorial policy Check list**

**nr Reporting summary**

**Author Check list**

**Supplementary Figures and Table**

Supplementary Figure1. Strategies for treatment of liver fibrosis.

Supplementary Figure 2. Stability of PRL.

Supplementary Figure 3. Survival study of PRL.

Supplementary Figure 4. Cell population analysis by qPCR in the liver tissue.

Supplementary Figure 5. Viability of cells treated with melittin.

Supplementary Figure 6. Gating strategy and FMO control for FAP-positive aHSC.

Supplementary Figure 7. In vivo antifibrotic effect of PRL treatment starting at week 4 of the $CCl_4$-induced liver fibrosis model.

Supplementary Table 1. Primer sets for quantitative real time-PCR .

**Source Data file**

Source Data file (Figure 2)

Source Data file (Figure 3)

Source Data file (Figure 4)

Source Data file (Figure 5)

Source Data file (Figure 6)

Source Data file (Figure 7)

Source Data file (Suppl Fig 2)

Source Data file (Suppl Fig 3)

Source Data file (Suppl Fig 4)

Source Data file (Suppl Fig 5)

Source Data file (Suppl Fig 7)
