# Supplementary material for: Fibroblast activation protein activated antifibrotic peptide delivery attenuates fibrosis in mouse models of liver fibrosis
Source: Nat Commun. 2022 Mar 21;13:1516. doi: 10.1038/s41467-022-29186-8 (PMC8938482; doi:10.1038/s41467-022-29186-8)
Supplement: Supplementary file 3 — Reporting Summary [file 41467_2022_29186_MOESM3_ESM.pdf]

nature research

Corresponding author(s): Yu-Kyoung Oh

Last updated by author(s): Feb 12, 2022

Reporting Summary

Nature Research wishes to improve the reproducibility of the work that we publish. This form provides structure for consistency and transparency in reporting. For further information on Nature Research policies, see our Editorial Policies and the Editorial Policy Checklist.

Statistics

For all statistical analyses, confirm that the following items are present in the figure legend, table legend, main text, or Methods section.

n/a	Confirmed	
☐	☒	The exact sample size (*n*) for each experimental group/condition, given as a discrete number and unit of measurement
☐	☒	A statement on whether measurements were taken from distinct samples or whether the same sample was measured repeatedly
☐	☒	The statistical test(s) used AND whether they are one- or two-sided<br>*Only common tests should be described solely by name; describe more complex techniques in the Methods section.*
☒	☐	A description of all covariates tested
☐	☒	A description of any assumptions or corrections, such as tests of normality and adjustment for multiple comparisons
☐	☒	A full description of the statistical parameters including central tendency (e.g. means) or other basic estimates (e.g. regression coefficient) AND variation (e.g. standard deviation) or associated estimates of uncertainty (e.g. confidence intervals)
☐	☒	For null hypothesis testing, the test statistic (e.g. F, t, r) with confidence intervals, effect sizes, degrees of freedom and P value noted<br>*Give P values as exact values whenever suitable.*
☒	☐	For Bayesian analysis, information on the choice of priors and Markov chain Monte Carlo settings
☒	☐	For hierarchical and complex designs, identification of the appropriate level for tests and full reporting of outcomes
☒	☐	Estimates of effect sizes (e.g. Cohen's *d*, Pearson's *r*), indicating how they were calculated

Our web collection on statistics for biologists contains articles on many of the points above.

Software and code

Policy information about availability of computer code

Data collection	Flow cytometry data were acquired with BD FACSDIVA™ (v8.0.1.).<br>LEICA Application Suite X (v3.6.0.) was used to collect confocal images.<br>Vectra (v3.0.5.) was used to collect tissue images.<br>Living image (v4.5.2.) was used to collect fluorescent images.<br>Applied Biosystems® 7300 Real-Time PCR System (v1.4.0.) was used to collect relative gene expression values.<br>ELSZ-1000 software (ver. 5.10) was used to measure size, zeta potential of particles.
Data analysis	Flow cytometry data were analyzed with FlowJo (v10).<br>LEICA Application Suite X (v3.6.0.) was used to analyze confocal images.<br>inForm (v2.4.11.) was used to analyze tissue images.<br>Living image (v4.5.2.) was used to analyze fluorescent images of mice.<br>GraphPad Prism (v8.0.) was used for statistical analysis.

For manuscripts utilizing custom algorithms or software that are central to the research but not yet described in published literature, software must be made available to editors and reviewers. We strongly encourage code deposition in a community repository (e.g. GitHub). See the Nature Research guidelines for submitting code & software for further information.

Data

Policy information about availability of data

All manuscripts must include a data availability statement. This statement should provide the following information, where applicable:

- Accession codes, unique identifiers, or web links for publicly available datasets
- A list of figures that have associated raw data
- A description of any restrictions on data availability

All datasets generated in the study are included in the manuscript, the supplementary information and source data. Source data for all figures are provided with this paper.

Field-specific reporting

Please select the one below that is the best fit for your research. If you are not sure, read the appropriate sections before making your selection.

☒ Life sciences ☐ Behavioural & social sciences ☐ Ecological, evolutionary & environmental sciences

For a reference copy of the document with all sections, see nature.com/documents/nr-reporting-summary-flat.pdf

Life sciences study design

All studies must disclose on these points even when the disclosure is negative.

Sample size	For in vitro studies, sample size was determined based on our own pilot studies and no statistically calculations were performed. Sample sizes are described in figure legends. For animal studies, sample sizes were calculated by performing a power analysis based on our previous study (Fan et al. Biomaterials. 2020), which have a power of 0.8 with type 1 error rates of 0.05. Sample sizes are described in figure legends.
Data exclusions	No data was excluded.
Replication	All experiments were conducted at least two times and could be reliably reproduced.
Randomization	Animals were randomly allocated into different experimental groups within each cage.<br>During the in vitro experiments, all samples randomly allocated into experimental groups.
Blinding	Investigators were blinded to group allocation during the sample preparation, sample treatment, data collection and analysis for in vitro and in vivo experiments.

Reporting for specific materials, systems and methods

We require information from authors about some types of materials, experimental systems and methods used in many studies. Here, indicate whether each material, system or method listed is relevant to your study. If you are not sure if a list item applies to your research, read the appropriate section before selecting a response.

Materials & experimental systems

n/a	Involved in the study	
☐	☒	Antibodies
☐	☒	Eukaryotic cell lines
☒	☐	Palaeontology and archaeology
☐	☒	Animals and other organisms
☒	☐	Human research participants
☒	☐	Clinical data
☒	☐	Dual use research of concern

Methods

n/a	Involved in the study	
☒	☐	ChIP-seq
☐	☒	Flow cytometry
☒	☐	MRI-based neuroimaging

Antibodies

Antibodies used

1. PE rat anti-mouse CD31 antibody (BioLegend, Clone 390, cat. No. 102407, San Diego, CA, USA) Lot. No. B261070
2. PE/Cyanine7 rat anti-mouse CD26 antibody (BioLegend, Clone H194-112, cat. No. 137810, San Diego, CA, USA) Lot. No. B292380
3. mouse anti-rat alpha-smooth muscle actin antibody (Abcam, Clone 1A4, cat. No. ab7817, Cambridge, UK) Lot. No. GR3356520-4
4. Alexa Fluor 594 goat anti-mouse IgG antibody(BioLegend, Clone Poly4053, cat. No. 405326, San Diego, CA, USA) Lot. No. B324994
5. APC rat anti-mouse CD26 antibody (BioLegend, Clone H194-112, cat. No. 137807, San Diego, CA, USA) Lot. No. B279349
6. PE rat anti-mouse CD31 antibody (Invitrogen, Clone 390, cat. No. 12-0311-82, Waltham, MA, USA) Lot. No. 2114546
7. PE rat anti-mouse F4/80 antibody (BioLegend, Clone BM8, cat. No. 123110, San Diego, CA, USA) Lot. No. B309222
8. Alexa Fluor 594 Rabbit anti-mouse alpha-smooth muscle actin antibody (Cell signaling technology, Clone D4K9N, cat. No. 36110S, Danvers, MA, USA) Lot. No. 1

9. Rabbit anti-mouse Fibroblast activation protein, alpha antibody (Abcam, Polyclonal, cat. No. ab28244, Cambridge, UK) Lot. No. GR217381-54
10. Rabbit anti-mouse Cytokeratin 7 antibody (Abcam, Clone EPR17078, cat. No. ab181598, Cambridge, UK) Lot. No. GR3321316-4
11. Alexa Fluor 647 goat anti-rabbit IgG antibody (Abcam, Polyclonal, cat. No. ab150083, Cambridge, UK) Lot. No. GR3370563-1
12. PerCP/Cy5.5-conjugated rat anti-mouse CD45 antibody (BioLegend, Clone 30-F11, cat. No. 103132, San Diego, CA, USA) Lot. No. B282872
13. PE-conjugated rat anti-mouse CD31 antibody (BioLegend, Clone 390, cat. No. 102407, San Diego, CA, USA) Lot. No. B261070

Validation

All antibodies were verified by the supplier and each lot has been quality tested. All the antibodies used are from commercial sources and have been validated by the vendors. Validation data are available on the manufacturer's website.
1. PE rat anti-mouse CD31 antibody: https://www.biolegend.com/en-us/products/pe-anti-mouse-cd31-antibody-122
2. PE/Cyanine7 rat anti-mouse CD26 antibody: https://www.biolegend.com/en-us/products/pe-cyanine7-anti-mouse-cd26-dpp-4-antibody-10368
3. mouse anti-rat alpha-smooth muscle actin antibody: https://www.abcam.com/alpha-smooth-muscle-Actin-antibody-1A4-ab7817.html?gclsrc=aw.ds|aw.ds&gclid=EAIaIQobChMIkLD_2PWR8gIVUrGWCh1D1QDcEAAYASAAEgIq6vD_BwE
4. Alexa Fluor 594 goat anti-mouse IgG antibody: https://www.biolegend.com/en-us/products/alexa-fluor-594-goat-anti-mouse-igg-minimal-x-reactivity-9706
5. APC rat anti-mouse CD26 antibody: https://www.biolegend.com/en-us/products/apc-anti-mouse-cd26-dpp-4-antibody-6947
6. PE rat anti-mouse CD31 antibody: https://www.thermofisher.com/antibody/product/CD31-PECAM-1-Antibody-clone-390-Monoclonal/12-0311-82
7. PE rat anti-mouse F4/80 antibody:https://www.biolegend.com/en-us/products/pe-anti-mouse-f4-80-antibody-4068
8. Alexa Fluor 594 Rabbit anti-mouse alpha-smooth muscle actin antibody: https://www.cellsignal.com/products/antibody-conjugates/a-smooth-muscle-actin-d4k9n-xp-rabbit-mab-alexa-fluor-594-conjugate/36110?site-search-type=Products&N=4294956287&Ntt=36110s&fromPage=plp&_requestid=1536978
9. Rabbit anti-mouse Fibroblast activation protein, alpha antibody: https://www.abcam.com/fibroblast-activation-protein-alpha-antibody-ab28244.html
10. Rabbit anti-mouse Cytokeratin 7 antibody: https://www.abcam.com/cytokeratin-7-antibody-epr17078-cytoskeleton-marker-ab181598.html
11. Alexa Fluor 647 goat anti-rabbit IgG antibody: https://www.abcam.com/goat-rabbit-igg-hl-alexa-fluor-647-preadsorbed-ab150083.html
12. PerCP/Cy5.5-conjugated rat anti-mouse CD45 antibody: https://www.biolegend.com/en-us/products/percp-cyanine5-5-anti-mouse-cd45-antibody-4264?GroupID=BLG6829
13. PE-conjugated rat anti-mouse CD31 antibody: https://www.biolegend.com/en-us/products/pe-anti-mouse-cd31-antibody-122?GroupID=BLG1566

Eukaryotic cell lines

Policy information about cell lines

Cell line source(s)

LX-2 human hepatic stellate cells (#SCC064, Sigma-Aldrich; provided by Professor Sang Geon Kim, College of Pharmacy, Dongguk University, Republic of Korea)
Chang cells (ATCC CCL-13; provided by Professor Mi-Ock Lee, College of Pharmacy and Research Institute of Pharmaceutical Sciences, Seoul National University, Republic of Korea)
Chang cells are listed in the ICLAC register as a 'misidentified cell line'.

Authentication

Cell lines were authenticated by periodic morphology check via microscope

Mycoplasma contamination

All cell lines were tested for mycoplasma contamination. No mycoplasma contamination was found.

Commonly misidentified lines
(See ICLAC register)

No commonly misidentified cell lines are used in this study.

Animals and other organisms

Policy information about studies involving animals; ARRIVE guidelines recommended for reporting animal research

Laboratory animals

All animals were female and 8 weeks old at the start of the experiment. Strain BALB/c was used throughout animal experiment in BDL induced liver fibrosis model (Rodent Chow; Cat# 38057, Purina Lab, Missouri, USA) And strain C57BL/6 was used throughout animal experiment in CCl4-induced liver fibrosis model and CDAHFD (Cat# A06071302, Research Diets, New Brunswick, NJ) induced liver fibrosis model. All mice were housed up to 5 per cage with a 12 hr light/dark cycle allowed ad libitum access to food and water under the ambient temperature of 23°C ± 2°C and humidity of 50% ± 10%.

Wild animals

No wild animal was used in this study.

Field-collected samples

No field-collected samples was used in this study.

Ethics oversight

All animals animals were maintained and used in accordance with Guidelines for the Care and Use of Laboratory Animals of the Institute of Laboratory Animal Resources, Institutional Animal Care and Use Committee of Seoul National University (Seoul, Republic of Korea; approved animal experimental protocol number, SNU-130129-3-1).

Note that full information on the approval of the study protocol must also be provided in the manuscript.

Flow Cytometry

Plots

Confirm that:

☒ The axis labels state the marker and fluorochrome used (e.g. CD4-FITC).

☒ The axis scales are clearly visible. Include numbers along axes only for bottom left plot of group (a 'group' is an analysis of identical markers).

☒ All plots are contour plots with outliers or pseudocolor plots.

☒ A numerical value for number of cells or percentage (with statistics) is provided.

Methodology

Sample preparation	Liver tissues were perfused with HEPES buffer (Sigma-Aldrich; cat. No. H4034) containing collagenase D (Sigma-Aldrich; cat. No. C5138-1G) and pronase (Sigma-Aldrich), and digested with stirring at 37°C for 30 min. Cells were collected by centrifugation at 580 xg for 10 min, and dead cells were excluded using a Zombie Red Fixable Viability Kit (BioLegend). The cells were then stained with PE/Cyanine7-conjugated rat anti-mouse CD26 antibody (BioLegend; cat. No. 137810), PE-conjugated rat anti-mouse CD31 antibody (BioLegend; cat. No. 102407, Lot. No. B261070), PerCP/Cy5.5-conjugated rat anti-mouse CD45 antibody (BioLegend; cat. No. 103132, Lot. No. B282872), and rabbit anti-mouse FAP antibody (Abcam; cat. No. ab28244, Lot No. GR217381-54) for 1 hr at 4°C. Secondary antibody staining was performed with Alexa Fluor 647-conjugated goat anti-rabbit IgG antibody (Abcam; cat. No. ab150083, Lot No. GR3370563-1) for 30 min at 4°C.
Instrument	LSRFortessa II, BD Biosciences, Becton Dickinson, NJ, USA
Software	Flowjo v10
Cell population abundance	Cell count of 10,000 events was collected of a relevant cell population after initial gating.
Gating strategy	Initial cell populations were gated for singlet and doublet cells using FSC-A/ FSC-W gating. FMO control stained cells were used to distinguish between background staining and specific antibody staining. Detailed gating strategies were provided within the Supplementary Figure 6.

☒ Tick this box to confirm that a figure exemplifying the gating strategy is provided in the Supplementary Information.
